# Supplementary material for: Living (stained) foraminifera in the Lesser Syrtis (Tunisia): influence of pollution and substratum
Source: PeerJ. 2020 Apr 6;8:e8839. doi: 10.7717/peerj.8839 (PMC7144591; doi:10.7717/peerj.8839)
Supplement: Supplemental Information S3 [file peerj-08-8839-s003.docx]

SYSTEMATIC OF BENTHIC FORAMINIFERA

Taxonomic list and synonymies of living (stained) benthic foraminifera from surface sediments (0-1cm) from the Gulf of Gabes and Djerba Island coast and Djerba lagoon.

Class FORAMINIFERA d'Orbigny, 1826

Suborder SACCAMMININA Lankester, 1885

Superfamily SACCAMMINACEA Brady, 1881

Family SACCAMMINIDAE Brady, 1884

Subfamily SACCAMMININAE Brady, 1884

Genus LAGENAMMINA Rhumbler, 1911

**Lagenammina fusiformis (Williamson, 1858)** Figure 6 (N, O)

1858 Proteonina fusiformis in Williamson, p. 1, pl. 1, fig. 1.

1947 Proteonina dusiformis (Williamson) in Höglundi, p. 52, pl. 4, fig. 21, text figs. 20, 21.

1960 Reophax fusiformis (Williamson) in Barker, pl. 30, figs. 7-10.

1970 Saccammina difflugiformis (Brady) in von Daniels, p. 66, pl. 1, figs. 2a, 2c.

1991 Lagenammina fusiformis (Williamson) in Cimerman and Langer, p. 15, pl. 1, figs. 4-5.

Superfamily HIPPOCREPINACEA Rhumbler, 1895

Family HIPPOCREPINIDAE Rhumbler, 1895

Subfamily HYPERAMMININAE Eimer and Fickert, 1899

Genus SACCORHIZA Eimer and Fickert, 1899

**Saccorhiza ramosa (Brady, 1879)**

1879a Hyperammina ramosa in Brady, p. 33, pl. 3, figs. 14, 15.

1884 Hyperammina ramosa in Brady, p. 261, pI. 23, figs. 15-19.

1910 Saccorhiza ramosa (Brady) in Cushman, p. 65, pl. 30, figs. 3, 4, text fig. 8.

1921 Saccorhiza ramosa (Brady) in Cushman, p. 54, pI. 4, fig. 5.

1982 Saccorhiza ramosa (Brady) in Tappan and Loeblich, pl. 47, fig. 2.

1994 Saccorhiza ramosa (Brady) in Loeblich Tappan, p. 14, pl. 1, figs. 4, 5.

Superfamily AMMODSCACEA Reuss, 1862

Family AMMODISCIDAE Reuss, 1862

Subfamily TOLYPAMMINIAE Cushman, 1928

Genus AMMOLAGENA Eimer and Fickert, 1899

**Glomospira gordialis (Jones and Parker, 1860)** Figure 6 (L, M)

1860 Trochammina squamata Jones and Parker var. gordialis in Jones and Parker, p.304.

1910 Gordiammina gordialis (Jones and Parker) in Cushman, p 76, figs. 98-100.

1918 Glomospira gordialis (Jones and Parker) in Cushman, p. 99, pl. 36, figs. 7-9.

1987 Glomospira gordialis (Jones and Parker) in Loeblich and Tappan, p. 50, pl. 38, figs. 5, 6.

1991 Glomospira gordialis (Jones and Parker) in Cimerman and Langer, p.17, pl. 3, figs. 4-5.

Subfamily AMMOVERTELLININAE Saidova, 1981

Genus GLOMOSPIRA Rzehak, 1885

**Glomospira charoides (Jones and Parker, 1860)** Figure 6 (J, K)

1860 Trochammina squamata var. charoides Jones and Parker in Jones and Paker, p. 304

1884 Ammodiscus charoides (Jones and Parker) in Brady, p. 334, pl. 36, figs. 10-16

1918 Glomospira charoides (Jones and Parker) in Cushman, p. 100, pl. 36, figs. 10-15

1947 Glomospira charoides (Jones and Parker) in Höglund, p. 129, pl. 3, fig. 11

1981 Glomospira charoides (Jones and Parker) in Resig, pl. 9, fig. 8

1987 Repmanina charoides (Jones and Parker) in Loeblich and Tappan, p. 52, pl. 39, figs. 24-26

1991 Repmanina charoides (Jones and Parker) in Cimerman and Langer, p. 17, pl. 3, figs. 6- 9

1994 Usbekistania charoides (Jones and Parker) in Jones, p. 43, pl. 38, figs. 10-16

2008 Glomospira charoides (Jones and Parker) in Abu-Zied et al., p. 51, pl. 1, figs. 2-3

2013 Glomospira charoides (Jones and Parker) in Holbourn et al., p. 268-269, fig. 1

2015 Glomospira charoides (Jones and Parker) in Spezzaferri et al., pl. 2, fig. 5

Order BULIMINIDA Fursenko, 1958

Superfamily BOLIVINACEA Glaessner, 1937

Family BOLIVINIDAE Glaessner, 1937

Genus ABDITODENTRIX Patterson, 1985

**Abditodentrix rhomboidalis (Millet, 1899)** Figure 9 (P, Q, R)

1899 Textularia rhomboidalis in Millet, p. 559, pl. 7, fig. 4 (fide Ellis and Messina, 1949).

1905 Textularia rhomboidalis Millet in Sidebottom, p. 8, pl. 2, fig. 2.

1922b Bolivina rhomboidalis (Millet) in Cushman, p. 44.

1970 Brizalina rhomboidalis (Millet) in Murray, p. 63, figs, 10 D, E.

1987 Bolivina rhomboidalis (Millet) in Baccaert, p. 180, pl. 73, figs. 3-4.

1991 Abditodentrix rhomboidalis (Millet) in Cimerman and Langer, p. 60, pl. 61, figs. 4-6.

Genus BOLIVINA d’Orbigny, 1839c

**Bolivina alata (Seguenza, 1862)**

1862 Vulvulina alata in Seguenza, p. 115, fig. 5a, b.

1937c Bolivina alata (Seguenza) in Cushman, p. 106, pl. 13, figs. 3-11.

1948 Bolivina alata (Seguenza) in Renz, p. 116, pl. 6, fig. 26, pl. 12, fig. 12.

**Bolivina cistina (Cushman, 1936)**

1936 Bolivina cistina Cushman: p. 55, pl. 8, fig. 4.

1990 Bolivina cistina (Cushman) in Hasegawa et al., p. 476, pl. 3, figs. 1, 2.

2012 Bolivina cistina (Cushman) in Milker and Schmiedl, p. 80, fig. 19.19-20.

**Bolivina difformis (Williamson, 1858)** Figure 8 (II, JJ)

1858 Textularia variabilis var. difformis in Williamson, p. 77, pl. 6, figs. 166, 167

2003 Bolivina difformis (Williamson) in Murray, p. 19, pl. 6, fig. 2

2012 Bolivina difformis (Williamson) in Boltovskoy et al., p. 17, pl. 2, figs. 16, 17

2015 Bolivina difformis (Williamson) in Spezzaferri et al., p. 73, pl. 19, fig. 6

**Bolivina pseudoplicata (Heron-Allen and Earland, 1930)**

1930 Bolivina pseudoplicata in Heron-Allen and Earland, p. 81, pl. 3, figs. 36-40.

1937c Bolivina pseudplicata (Heron-Allen and Earland) in Cushman, p. 166, pl. 19, figs. 12-20.

1971 Bolivina pseudoplicata (Heron-Allen and Earland) in Murray, p. 107, pl. 43, figs. 1-7.

1991 Bolivina pseudoplicata (Heron-Allen and Earland) in Cimerman and Langer, p. 58, pl. 61, figs. 1-3.

**Bolivina pseudopunctata (Höglund, 1947)**

1947 Bolivina pseudopunctata in Höglund, p. 273, pl. 24, figs, 23-24

1971 Brizalina pseudopunctata (Höglund) in Murray, p. 109, pl. 44, figs. 3-6

2003 Bolivinellina pseudopunctata (Höglund) in Murray, p. 20, pl. 6, fig. 1

**Bolivina plicatella (Cushman, 1930)** Figure 8 (GG, HH)

1930 Bolivina plicatella in Cushman, p. 46, pl. 18, fig. 10a, b.

1931 Bolivina plicatella (Cushman) in Cushman and Parker, p. 15, pl. 3, fig. 19.

1965 Bolivina plicatella plicatella (Cushman) in Souaya, p. 323, pl. 2, fig. 22.

1990 Bolivina plicatella (Cushman) in Hasegawa et al., p. 476, pl. 3, figs. 3, 4.

1993 Bolivina plicatella plicatella (Cushman) in Mehrnusch, p. 11, figs. 22-27.

2012 Bolivina plicatella (Cushman) in Milker and Schmiedl, fig. 19.21.

**Bolivina pseudoplicata (Heron-Allen and Earland, 1930)**

1930 Bolivina pseudoplicata in Heron-Allen and Earland pp. 81-82, pl. 3, figs. 36-40.

1958 Bolivina pseudoplicata (Heron-Allen and Earland) in Parker, p. 261, pl. 2, fig. 8.

1960 Bolivina pseudoplicata (Heron-Allen and Earland) in Hofker, p. 251, pl. D, fig. 108.

1990 Bolivina pseudoplicata (Heron-Allen and Earland) in Hasegawa et al., p. 476, pl. 3, figs. 5, 6.

1991 Bolivina pseudoplicata (Heron-Allen and Earland) in Cimerman and Langer, p. 58, pl. 61, figs. 1, 2.

1991 Bolivina pseudoplicata (Heron-Allen and Earland) in Rasmussen, p. 363, fig. 6, no. 11.

1993 Bolivina pseudoplicata (Heron-Allen and Earland) in Sgarrella and Moncharmont Zei, p. 208, pl. 14, figs. 9, 10.

2003 Bolivina pseudoplicata (Heron-Allen and Earland) in Murray, p. 19, fig. 5, no. 17.

2005 Bolivina pseudoplicata (Heron-Allen and Earland) in Rasmussen, p. 80, pl. 9, figs. 16, 17.

2012 Bolivina pseudoplicata (Heron-Allen and Earland) in Milker and Schmiedl, fig. 19.22-23.

**Bolivina subspinescens (Cushman, 1922a)**

1922a Bolivina subspinescens in Cushman, p. 48, pl. 7, fig. 5.

1985 Bolivina subspinescens (Cushman) in Hermelin and Scott, p. 204, pl. 3, fig. 2.

1990 Bolivina subspinescens (Cushman) in Hasegawa et al., p. 476, pl. 3, fig. 10.

1992 Bolivina subspinescens (Cushman) in Schiebel, p. 34, pl. 1, fig. 5.

1993 Bolivina subspinescens (Cushman) in Sgarrella and Moncharmont Zei, p. 210, pl. 14, figs. 12, 13.

2005 Bolivina subspinescens (Cushman) in Debenay et al., p. 336 pl. 3, fig. 9.

2005 Sagrina subspinescens (Cushman) in Rasmussen, p. 85, pl. 11, fig. 2.

2008 Bolivina subspinescens (Cushman) in Leiter, p. 24, pl. 3, fig. 4.

2012 Bolivina subspinescens (Cushman) in Milker and Schmiedl, Fig. 19.24.

**Bolivina variabilis (Williamson, 1858)**

1858 Textularia variabilis in Williamson, p. 76, pl. 6, figs. 162, 163.

1937c Bolivina variabilis (Williamson) in Cushman, p. 158, pl. 16, figs. 6, 12-14.

1942 Bolivina variabilis (Williamson) in Cushman, p. 32, pl. 9, fig. 3.

1971 Brizalina variabilis (Williamson) in Murray, p. 113, pl. 46, figs. 1-3.

1972 Bolivina variabilis (Williamson) in Rosset-Moulinier, p. 161, pl. 9, fig. 13.

1991 Bolivina variabilis (Williamson) in Cimerman and Langer, p. 59, pl. 61, figs. 7-8.

Genus BRIZALINA Costa, 1856

**Brizalina earlandi (Parr, 1950)**

1950 Bolivina earlandi in Parr, pl. 12, fig. 16 a, b.

1960 Bolivina earlandi (Parr) in Barker, pl. 52, figs. 18, 19.

1991 Brizalina earlandi (Parr) in Cimerman and Langer, p. 59, pl. 62, figs. 11-13.

**Brizalina spathulata (Williamson, 1858)**

1858 Textularia variabilis var. spathulata in Williamson, p. 76, pl. 6, figs. 164, 165.

1937a Bolivina spathulata (Williamson) in Cushman, p. 162, pl. 15, figs. 20-24.

1974 Brizalina spathulata (Williamson) in Colom, p. 121, fig. 18 h, i.

1991 Brizalina spathulata (Williamson) in Cimerman and Langer, p. 60, pl. 62, figs. 3-5.

**Brizalina striatula (Cushman, 1922)** Figure 8 (KK, LL)

1922a Bolivina striatula in Cushman, p. 27, pl. 3, fig. 10 (fide Ellis and Messina, 1940).

1922b Bolivina striatula (Cushman) in Cushman, p. 43.

1937c Bolivina striatula (Cushman) in Cushman, p. 154, pl. 18, figs. 30, 31.

1974 Bolivina striatula (Cushman) in Colom, p. 120, fig. 17 a-g.

**Brizalina variabilis (Williamson), 1858**

1858 Textularia variabilis in Williamson, p. 76, pl. 6, l1gs. 162,163.

1937c Bolivina variablis (Williamson) in Cushman, p. 158, pl. 16, figs. 6, 12-14.

1971 Brizalina variabilis (Willamson) in Murray, p. 113. pl. 46, figs. 1-3.

1992 Bolivina subexcavata Cushman and Wickenden in Hatta and Ujiié (non Cushman and Wickenden. 1929), p. 171, pl. 25, figs. 2, 3.

Genus GLOBOCASSIDULINA Voloshinova, 1960

**Globocassidulina subglobosa (Brady, 1884)**

1884 Cassidulina subglobosa in Brady, p. 430, pl. 54, fig. 17 a-c.

1922b Cassidulina subglobosa (Brady) in Cushman, p. 127, pl. 24, fig. 6.

1972 Cassidulina subglobosa (Brady) in Rosset-Moulinier, p. 185, pl. 11, fig. 20.

1991 Globocassidulina subglobosa (Brady) in Cimerman and Langer, p. 61, pl. 63, figs. 4-6.

Superfamily BULIMINACEA Jones, 1875

Family BULIMINIDAE Jones, 1875

Genus BULIMINA d’Orbigny, 1826

**Bulimina aculeata (d’Orbigny, 1826)**

1826 Bulimina aculeata in d’Orbigny, p. 269, no. 7.

1922b Buliminia aculeata (d’Orbigny) in Cushman, p. 96, pl. 22, figs. 1,2.

1960 Bulimina aculeata (d’Orbigny) in Baker, pl. 51, figs. 7-9.

1970 Bulimina aculeata (d’Orbigny) in von Daniels, p. 82, pl. 5, fig. 8.

1974 Bulimina aculeata (d’Orbigny) in Colom, p. 115, fig. 16 g.

**Bulimina cf. B. alazanensis (Cushman, 1927)**

1927 cf. Bulimina alazanensis in Cushman, p. 161, pl. 25, fig. 4.

1971 Bulimina cf. B. alazanensis (Cushman) in Murray, p. 115, pl. 47, figs. 1-7.

1991 Bulimina cf. B. alazanensis (Cushman) in Cimerman and Langer, p. 62, pl. 64, figs. 1-2.

**Bulimina elongata (d’Orbigny, 1846)** Figure 9 (C, D)

1846 Bulimina elongata in d’Orbigny, p. 187, pl. 11, figs. 19,20.

1922b Bulimina elongata (d’Orbigny) in Cushman, p. 107.

1960 Bulimina elongata (d’Orbigny) in Baker, pl. 51, figs. 1,2.

1972 Bulimina elongata (d’Orbigny) in Rousset-Moulinier, p. 163, pl. 9, fig. 18.

1974 Bulimina elongata (d’Orbigny) in Colom, p. 116, fig. 16n.

1991 Bulimina elongata (d’Orbigny) in Cimerman and Langer, p. 62, pl. 64, figs. 3-8.

**Bulimina gibba (Fornasini, 1901)**

1884 Bulimina elegans (d’Orbigny) in Brady, p. 398, pl. 50, figs. 1, 2.

1901 Bulimina gibba in Fornasini, p. 378, figs. 32, 34.

1958 Bulimina gibba (Fornasini) in Parker, p. 261, pl. 2, figs. 21, 22.

1960 Bulimina gibba (Fornasini) in Hofker, p. 248, pl. D, figs. 91-95.

1994 Bulimina gibba (Fornasini) in Jones, p. 54, pl. 50, figs. 1, 2 [cop. Brady, 1884, figs. 1, 2].

2005 Bulimina gibba (Fornasini) in Rasmussen, p. 86, pl. 11, fig. 5.

2009 Bulimina gibba (Fornasini) in Milker et al., p. 216, pl. 2, fig. 23.

2012 Bulimina gibba (Fornasini) in Milker and Schmiedl, p. 88, fig. 20.22.

**Bulimina marginata (d’Orbigny, 1826)** Figure 9 (E, F)

1826 Bulimina marginata in d’Orbigny, p. 269, pl. 12, figs, 10-12.

1979 Bulimina marginata (d’Orbigny) in Hageman, p. 90, pl. 2, fig. 7.

1984 Bulimina aculeata in Vénec-Peyré (not d’Orbigny), in Ecomed, l. 6, fig. 2.

1987 Bulimina marginata forma marginata (d’Orbigny) in Jorissen, p. 46, pl. 4, figs. 6°, b.

1988 Bulimina marginata forma marginata (d’Orbigny) in Jorissen, p. 75, pl. 4, fig. 6, pl. 12, figs. 7, 14-17, pl. 13, figs. 5, 10, 11, 13-15, pl. 14, figs. 1-3, 7, 9-11, pl. 15, figs. 1,2.

1991 Bulimina marginata (d’Orbigny) in Cimerman and Langer, p. 62, pl. 64, figs, 9-11.

Genus GLOBOBULIMINA Cushman, 1927

**Globobulimina affinis (d’Orbigny, 1839a)**

1839a Bulimina affinis in d’Orbigny, p. 105, pl. 2, figs. 25, 26.

1993 Globobulimina affinis (d’Orbigny) in Sgarrella and Moncharmont Zei, p. 212, pl. 15, figs. 8, 9.

2005 Globobulimina affinis (d’Orbigny) in Rasmussen, p. 87, pl. 11, fig. 7.

2006 Globobulimina affinis (d’Orbigny) in Avsar et al., p. 133, pl. 2, fig. 6.

2008 Globobulimina affinis (d’Orbigny) in Abu Zied et al., p. 52, pl. 2, figs. 13, 14.

2008 Globobulimina affinis (d’Orbigny) in Leiter, p. 40, pl. 4, fig. 4.

2009 Globobulimina affinis (d’Orbigny) in Milker et al., p. 218, pl. 3, fig. 1.

2012 Globobulimina affinis (d’Orbigny) in Milker and Schmiedl, p. 89, fig. 20.24.

Genus PROTOGLOBOBULIMINA Hofker, 1951

**Protoglobobulimina pupoides (d’Orbigny, 1846)**

1846 Bulimina pupoides in d’Orbigny, p. 185, pl. 11, figs. 11-12.

1987 Protoglobobulimina pupoides (d’Orbigny) in Loeblich and Tappan, p. 522, pl. 572, figs. 1-6.

1991 Protoglobobulimina pupoides (d’Orbigny) in Cimerman and Langer, p. 62, pl. 65, figs. 1-3.

Family BULIMINELLIDAE Hofker, 1951

Genus BULIMINELLA Cushman, 1911

**Buliminella elegantissima (d’Orbigny, 1839)** Figure 9 (A, B)

1839 Bulimina elegantissima in d’Orbigny, p. 51, pl. 7, figs. 13, 14.

1922 Buliminella elegantissima (d’Orbigny) in Cushman 1922, p. 108.

1971 Buliminella elegantissima (d'Orbigny) in Murray, p. 105, pl. 42, figs. 1-4.

2008 Buliminella elegantissima (d’Orbigny) in Vázquez Riveiros et al., p. 23, figs. 10.2a-10.2d.

Family SIPHOGENERINOIDIDAE Saidova, 1981

Subfamily TUBULOGENERININAE Saidova, 1981

Genus SIPHOGENERINA Schlumberger, 1882

**Siphogenerina raphana (Parker and Jones, 1865)** Figure 9 (K, L, M)

1865 Uvigerina (Sagrina) raphanus in Parker and Jones, p. 364, pI. 18, figs. 16, 17.

1884 Sagrina raphanus (Parker and Jones) in Brady (part), p. 585, pI. 75, figs. 2 L 22, 24 (not fig. 23).

1926 Siphogenerina raphanus (Parker and Jones) in Cushman (part), p. 4, pl. 1, figs. 1-4 (not pI. 5. figs. 1, 2.

1964 Siphogenerina raphanus (Parker and Jones) in Leroy, p. F-35, pl. 3. fig. 35, pl. 16, fig. 9.

1942 Siphogenerina raphana (parker and Jones) in Cushman, p. 55, pl. 15, figs. 6-9.

1990 Rectobolivina raphanus (Parker and Jones) in Akimoto, p. 209, pl. 15, fig. 6, pl. 16, fig. 4.

1992 Rectobolivina raphana (Parker and Jones) in Hatta and Ujiie, p. 174, pI. 26, figs. 11, 12.

1994 Siphogenerina raphana (Parker and Jones) in Loeblich and Tappan, p. 123, pl. 240, figs. 1-11.

Family UVIGERINIDAE Haeckel, 1894

Subfamily UVIGERININAE Haeckel, 1894

Genus UVIGERINA d’Orbigny, 1826

**Uvigerina canariensis (d’Orbigny, 1839)** Figure 9 (V, W)

1839 Uvigerina canariensis in d’Orbigny, p. 138, pl. I, figs. 25-27.

1884 Uvigerina canariensis in Brady, pl. LXXIV, figs. 1-3.

1898 Uvigerina canariensis in Bagg, pp. (31,32), 325, 326.

**Uvigerina mediterranea (Hofker, 1932)**

1932 Uvigerina mediterranea in Hofker, p. 118, textfig. 32 a-g

1974 Uvigerina mediterranea (Hofker) in Colom, p. 122, fig. 19 h-n

1991 Uvigerina mediterranea (Hofker) in Cimerman, p. 63, pl. 65, fig. 7-9

Subfamily ANGULOGERININAE Galloway, 1933

Genus ANGULOGERINA Cushman, 1927

**Angulogerina angulosa (Williamson, 1858)**

1858 Uvigerina angulosa in Williamson, p. 67, pl. 5, fig. 140.

1958 Angulogerina angulosa (Williamson) in Le Calvez, p. 180.

1960 Trilarina angulosa (Williamson) in Barker, pl. 74, figs. 15, 16.

1970 Trifarina angulosa (Williamson) in von Daniels, p. 83, pl. 6, fig. 4.

1979 Trifarina angulosa (Williamson) in Alfirevic, p. 121, pl. 25, fig. 3.

1987 Angulogerina angulosa (Williamson) in Loeblich and Tappan, p. 525, pl. 574, figs. 5-9.

1991 Angulogerina angulosa (Williamson) in Cimerman and Langer, p. 63, pl. 66, figs. 3-4.

Family REUSSELLIDAE Cushman, 1933b

Genus REUSSELLA Galloway, 1933

**Reussella spinulosa (Reuss, 1850)**

1850 Verneuillna spinulosa in Reuss, p. 374, pl. 47, fig. 12.

1970 Reussella spinulosa (Reuss) in von Daniels, p. 83, pl. 6, fig. 2.

1979 Reussella spinulosa (Reuss) in Alfirevic, p. 117, pl. 25, fig. 2.

1987 Reussella spinulosa (Reuss) in Loeblich and Tappan, p. 527, pl. 575, figs. 9-12.

1991 Reussella spinulosa (Reuss) in Cimerman and Langer, p. 63, pl. 66, figs. 5-8.

Superfamily FURSENKOINACEA Loeblich and Tappan, 1961

Family FURSENKOINIDAE Loeblich and Tappan, 1961

Genus FURSENKOINA Loeblich and Tappan, 1961

**Fursenkoina acuta (d'Orbigny, 1846)** Figure 9 (I, J)

1846 Polymorphina acuta in d'Orbigny, p. 234, pl. 13, figs. 4-5.

1848 Virgulina schrcibersiana in Czjzek, p. 147, pl. 13, figs. 18-21.

1972 Virgulina schreibersiana (Czjzek) in Rosset-Moulinier, p. 184.

1985 Fursenkoina acuta (d'Orbigny) in Papp and Schmid, p. 82, pl. 75, figs. 1-6.

1991 Fursenkoina acuta (d'Orbigny) in Cimerman and Langer, p. 64, pl. 67, figs. 1-2.

Genus SIGMAVIRGULINA Loeblich and Tappan, 1957

**Sigmavirgulina tortuosa (Brady, 1881)** Figure 9 (N, O)

1881 Bolivina tortuosa in Brady, p. 57.

1884 Bolivina tortuosa (Brady) in Brady, p. 420, pl. 52, figs. 31-34.

1931 Bolivina tortuosa (Brady) in Cushman and Parker, p. 16, pl. 3, fig. 22.

1987 Sigmavirgulina tortuosa (Brady) in Loeblich and Tappan, p. 153, pl. 579, figs. 1-5.

1994 Sigmavirgulina tortuosa (Brady) in Jones, p. 58, pl. 52, figs. 31-34 [cop. Brady, 1884, figs. 31-34].

2012 Sigmavirgulina tortuosa (Brady) in Milker and Schmiedl, p. 93, fig. 21.12.

Order LITUOLIDA Lankester, 1885

Suborder HORMOSININA Mikhalevich, 1980

Superfamily HORMOSINACEA Haeckel, 1894

Family REOPHACIDAE Cushman, 1910

Subfamily REOPHACINAE Cushman, 1910

Genus REOPHAX de Montfort, 1808

**Reophax scorpiurus (Montfort, 1808)**

1808 Reophax scorpiurus in Montfort, p. 331 (fide Ellis and Messina, 1940).

1920 Reophax scorpiurus (Montfort) in Cushmann, p. 6, pl. 1, figs. 5-7.

1970 Reophax scorpiurus (Montfort) in von Daniels, p. 68, pl. 1, fig. 10.

1971 Reophax scorpiurus (Montfort) in Murray, p. 19, pl. 2, figs. 5-7.

1974 Reophax scorpiurus (Montfort) in Colom, p. 86, figs. 5 k, 1.

1979 Reophax scorpiurus (Montfort) in Alfirević, p. 59, pl. 1, fig. 4.

1987 Reophax scorpiurus (Montfort) in Loeblich and Tappan, p. 58, pl. 44, figs. 1-3.

1991 Reophax scorpiurus (Montfort) in Cimerman and Langer, p. 17, pl. 4, figs. 1-4.

Suborder LITUOLINA Lankester, 1885

Superfamily LITUOLACEA de Blainville, 1827

Family DISCAMMINIDAE Mikhalevich, 1980

Genus AMMOSCALARIA Höglund, 1947

**Ammoscalaria runiuina (Heron-Allen and Earland, 1916)** Figure 6 (C, D)

1916 Haplophragmium runiannum in Heron-Allen and Earland, p. 224, figs. 15-18 (fide Ellis and Messina, 1940).

1947 Ammoscalaria runiana (Heron-Allen and Earland) in Höglund, pl. 9, figs. 23, 24.

1971 Ammoscalaria runiana (Heron-Allen and Earland) in Murray, p. 29, pl. 7, figs. 6-8.

1991 Ammoscalaria runiuina (Heron-Allen and Earland) in Cimerman and Langer, p. 19, pl. 5, figs. 7-8.

Suborder LITUOLINA Lankester, 1885

Superfamily LITUOLACEA de Blainville, 1827

Family LITUOLIDAE de Blainville, 1827

Subfamily AMMOMARGINULININAE Podobina, 1978

Genus AMMOBACULITES Cushman, 1910

**Ammobaculites agglutinans (d’Orbigny, 1846)**

1846 Spirolina agglutinans d'Orbigny in d'Orbigny, p. 137, pl. 7, figs. 10-12.

1884 Haplophragmium agglutinans (d’Orbigny) in Brady, p. 301, pl. 32, figs. 19-20, 24-26.

1986 Ammobaculites agglutinans (d’Orbigny) in Schröder, p. 50, pl. 21, figs 1-4.

1988 Ammobaculites agglutinans (d’Orbigny) in Zheng, p. 66, pl. 23, fig. 7.

1994 Ammobaculites agglutinans (d’Orbigny) in Jones, p. 39, pl. 32, figs. 19-20, 24-26.

Suborder SPIROPLECTAMMININA Mikhalevich 1980

Superfamily SPIROPLECTAMMINACEA Cushman, 1927

Family SPIROPLECTAMMINIDAE Cushman, 1927

Subfamily SPIROPLECTAMMININAE Cushman, 1927

Genus SPIROPLECTINELLA Kisel’man, 1972

**Spiroplectinella sagittula (d’Orbigny, 1839)**

1939b Texctularia sagittula in d’Orbigny, p. 138, pl. 1, figs. 19-21.

1933 Textularia sagittula Defrance in Lacroix, p. 1, textfigs. 1-8.

1958 Textularia sagittula d’Orbigny in Le Calvez, p. 150.

1974 Textularia sagittula d’Orbigny in Le Calvez, p. 82, pl. 21, figs. 1-5.

1987 Textularia sagittula Defrance in Loeblich and Tappan, p., 173, pl. 193, figs. 1, 2.

1991 Spiroplectinella sagittula (d’Orbigny, 1939) in Cimerman and Langer, p. 19, figs. 5-6.

Genus SPIROPLECTAMMINA Cushman, 1927

**Spiroplectammina earlandi (Parker, 1952)**

1932 Textularia elegans in Lacroix, p. 14, fig. 11.

1932 Textularia elegans in Lacroix, p. 8, fig. 4-6.

1933 Textularia tenuissima in Earland, p. 95, pl. 3, figs. 21-30.

1952 Textularia earlandi in Parker, p. 458, pl. fig. 4-5.

1989 Spiroplectammina earlandi (Parker) in Bender, 298, pl. 16, fig. 24.

1993 Spiroplectammina earlandi (Parker) in Hottinger et al., p. 31, pl. 6, figs. 5-10.

Suborder TROCHAMMININA Saidova, 1981

Superfamily TROCHAMMINACEA Schwager, 1877

Family TROCHAMMININAE Schwager, 1877

Subfamily TROCHAMMININAE Schwager, 1877

Genus AMMOGLOBIGERINA Eimer and Fickert, 1899

**Ammoglobigerina globigeriniformis (Parker and Jornes, 1865)** Figure 6 (A, B)

1865 Lituola nautiloidea Lamarck var. globigeriniformis in Parker and Jones, p. 407, pl. 15, figs 46, 47, pl. 17, figs. 96-98 (fide Ellis and Messina, 1940).

1960 Ammoglobigerina globigeriniformis (Parker and Jones) in Barker, pl. 35, figs. 10, 11.

1987 Ammoglobigerina globigeriniformis (Parkerand Jones) in Loeblich and Tappan, p. 120, pl. 128, figs. 9, 10.

1991 Ammoglobigerina globigeriniformis (Parker and Jornes, 1865) in Cimerman and Langer, p.20, pl. 7, figs. 4-6.

Genus PARATROCHAMMINA Brönnimann, 1979

**Paratrochammina challengeri (Brönnimann and Whittaker, 1987)** Figure 6 (P, Q)

1884 Haplophragmium globigeriniforme (Parker and Jones) in Brady (part, non Lituolidea nautiloidea var. globigeriniformis Parker and Jones, 1865), p. 312, pI. 35, fig. 10 (not fig. II).

1987 Paratrochammina challengeri in Bronnimann and Whittaker, p. 43, figs. 16H-K.

1988 Trochammina globigeriniformis (Parker and Jones) in Zheng (non Parker and Jones, 1865), p. 82. pl. 40, fig. 6.

1994 Paratrochammina challengeri (Bronnimann and Whittaker) in Loeblich and Tappan, p. 23, pl. 22, figs. 7-12.

**Paratrochammina madeirae (Brönnimann, 1979)** Figure 6 (R, S)

1979 Paratrochammina madeira in Brönnimann, p. 7, pl. 7, figs. a-c,f , h, pl. 10, figs. b, e.

1993 Paratrochammina madeira (Brönnimann) in Hottinger et al., p. 32, pl. 7, figs. 11-15.

Genus TROCHAMMINA Parker and Jones, 1859

**Trochammina inflata (Montagu, 1803)** Figure 6 (T, U)

1803 Nautilus inflatus in Montagu, p. 81 (fide Ellis and Messina, 1940).

1960 Trochammina inflata (Montagu) in Barker, pl. 41, fig. 4.

1972 Trochammina inflata (Montagu) in Rosset-Moulinier, p. 122, pl. 3, figs. 11, 12.

1987 Trochammina inflata (Montagu) in Loeblich and Tappan, p. 122, pl. 129, figs. 20-23.

1991 Trochammina inflata (Montagu) in Cimerman and Langer, p. 20, pl. 7, figs. 7-9.

Subfamily POLYSTOMAMMININAE Brönnimann and Beurlen, 1977

Genus DEUTERAMMINA Brönnimann, 1976

**Deuterammina dublinensis (Brönnimann and Whittaker, 1983)**

1983 Deuterammina dublinensis in Brönnimann and Whittaker, p. 353.

1987 Deuterammina dublinensis (Brönnimann and Whittaker) in Loeblich and Tappan, p. 131, pl. 135, figs. 1-5.

Order TEXTULARIIDA Delage and Herouard, 1896

Suborder TEXTULARIINA Delage and Herouard, 1896

Superfamily LITUOLACEA de Blainville, 1827

Family HAPLOPHRAGMOIDIDAE Maync, 1952

Genus CRIBROSTOMOIDES Cushman, 1910

**Cribrostomoides jeffreysii (Williamson, 1858)**

1858 Nonionina jeffreysii in Williamson, p. 34, pl. 3, figs. 72, 73.

1884 Haplophragmium canariense (d'Orbigny, sp.) in Brady, 1884, p. 310, pl. 35, figs.1-3, 5.

1991 Cribrostomoides jeffreysii (Williamson) in Alberola et al., p. 80, pl. 1, figs. 1, 5.

1991 Labrospira kosterensis (Hoeglund) in Cimerman and Langer, p. 18, pl. 4, figs. 11-13.

1992 Labrospira jeffreysii (Williamson) in Schiebel, p. 17, pl. 7, fig. 4.

1992 Cribrostomoides jeffreysii (Williamson) in Wollenburg, p. 27, pl. 5, figs. 1, 4.

1993 Labrospira jeffreysii (Williamson) in Hottinger et al., p. 29, pl. 2, figs. 5-9.

1993 Cribrostomoides jeffreysii (Williamson) in Sgarrella and Moncharmont Zei, p. 157, pl. 2, figs. 8, 9.

1994 Veleroninoides jeffreysii (Williamson) in Jones, p. 41, pl. 35, figs. 1-3, 5 [cop. Brady, 1884, figs. 1-3, 5].

2003 Cribrostomoides jeffreysii (Williamson) in Murray, p. 11, fig. 2, no. 5.

2005 Cribrostomoides jeffreysii (Williamson) in Debenay et al., p. 332, pl. 1, fig. 4.

2012 Cribrostomoides jeffreysii (Williamson) in Milker and Schmiedl, p. 32, fig. 9.9-10.

Superfamily TEXTULARIACEA Ehrenberg, 1838

Family EGGERELLIDAE Cushman, 1937b

SubFamily EGGERELLINAE Cushman, 1937b

Genus EGGERELLA Cushman, 1933a

**Eggerelloides scaber (Williamson, 1858)** Figure 6 (H, I)

1858 Bulimina scabra in Williamson, p. 65, pl. 5, figs. 136, 137.

1922b Bulimina scabra (Williamson) in Cushman, p. 55.

1937b Eggerella scabra (Williamson) in Cushman, p. 50, pl. 5, fig. 10.

1960 Eggerella scabra (Williamson) in Barker, pl. 47, figs. 15-17.

1987 Eggerelloides scabrus (Williamson) in Loeblich and Tappan, p. 170, pl. 189, figs 5-7.

1991 Eggerelloides scabrus (Williamson) in Cimerman and Langer, p. 21, pl. 8, fig. 7.

2013 Eggerelloides scaber (Williamson) in Schönfeld et al., figs. 2d-f.

Family PSAMMOSPHAERIDAE Haeckel, 1894

Subfamily PSAMMOSPHAERINAE Haeckel, 1894

Genus PSAMMOSPHAERA Schulze, 1875

**Psammosphaera fusca (Schulze, 1875)** Figure 6 (V, W)

1875 Psammosphaera fusca in Schulze, p. 113, pl. 2, fig. 8 a-f.

1910 Psammosphaera fusca (Schulze) in Cushman, p. 35-36, figs. 25-28.

1910 Psammosphaera parva (Flint) in Cushman, pp. 36-37, figs. 29, 30.

1931 Psammosphaera fusca (Schulze) in Wiesner, p. 79, pl. 4, fig. 32.

1987 Psammosphaera fusca (Schulze) in Loeblich and Tappan, p. 6, pl. 19, figs. 2, 3.

1993 Psammosphaera fusca (Schulze) in Sgarrella and Moncharmont Zei, p. 151, pl. 1, fig. 14.

1994 Psammosphaera fusca (Schulze) in Jones, p. 31, pl. 18, figs. 1-8.

2005 Psammosphaera fusca (Schulze) in Rasmussen, p. 54, pl. 1, fig. 1.

2012 Psammosphaera fusca (Schulze) in Milker and Schmiedl, p. 26, fig. 9.2-3.

Family TEXTULARIIDAE Ehrenberg, 1838

Subfamily TEXTULARIINAE Ehrenberg, 1838

Genus TEXTULARIA Defrance, 1824

**Textularia agglutinans (d’Orbigny, 1839)** Figure 6 (DD, EE)

1839b Textularia agglutinans in D’Orbigny, p. 136, pl. 1, figs. 17, 18, 32-34.

1896 Textularia agglutinans (d’Orbigny) in Deželić, p. 76.

1932 Textularia agglutinans (d’Orbigny) in Lacroix, p. 16, fig. 14.

1958 Textularia agglutinans (d’Orbigny) in Le Calvez, p. 151, pl. 1, fig. 3.

1977 Textularia agglutinans (d’Orbigny) in Le Clavez, Y., p. 13, figs. 1-3.

1979 Textularia agglutinans (d’Orbigny) in Alfirević, p. 61, pl. 3, fig. 1.

1991 Textularia agglutinans (d’Orbigny) in Cimerman and Langer, p. 21, pl. 10, figs. 1-2.

**Textularia bocki (Höglund, 1947)**

1947 Textularia bocki in (Höglund), p. 171, pl. 12, figs. 5,6.

1932 Textularia agglutinans (d’Orbigny) in Lacroix, p. 16, fig. 13.

1958 Textularia bocki (Höglund) in Le Calvez, p. 150, pl. 1, fig. 4.

1991 Textularia bocki (Höglund) in Cimerman and Langer, p. 21, pl. 10, figs. 3-6.

**Textularia calva (Lalicker, 1935)** Figure 6 (FF, GG, HH)

1935 Textularia calva in Lalicker in p. 1, pl. 1, figs. 1, 2.

1940 Textularia calva (Lalicker) in Lalicker and McCulloch, p. 120, pl. 13, fig. 6.

1958 Textularia calva (Lalicker) in Parker, p. 254, pl. 1, fig. 4.

1991 Textularia bocki (Hoeglund) in Cimerman and Langer, p. 21, pl. 10, fig. 6.

1993 Textularia calva (Lalicker) in Sgarrella and Moncharmont Zei, p. 164, pl. 3, fig. 11.

2002 Textularia bocki (Hoeglund) in Kaminski et al., p. 170, pl. 1, figs. 1, 2.

2005 Textularia gramen (d’Orbigny) in Rasmussen, p. 56, pl. 1, fig. 17.

2009 Textularia calva (Lalicker) in Milker et al., p. 215, pl. 1, fig. 12.

2012 Textularia calva (Lalicker) in Milker and Schmiedl, p. 39, fig. 10.17.

**Textularia conica (d’Orbigny, 1839)** Figure 6 (Z, AA)

1839a Textularia conica in d’Orbigny, p. 143, pl. 1, figs. 19, 20.

1899 Textularia conica (d’Orbigny) in Flint, p. 285, pl. 29, fig. 6.

1932 Textularia conica (d’Orbigny) in Cushman, p. 11, pl. 2, figs. 8-11.

1977a Textularia conica (d’Orbigny) in Le Calvez, p. 18, figs. 1, 2.

1991 Textularia conica (d’Orbigny) in Cimerman and Langer, p. 22, pl. 10, figs. 7-9.

**Textularia pala (Czjzek, 1848)**

1848 Textularia pala in Czjzek, p. 148, pl. 13, figs. 25-27.

1991 Textularia truncata (Höglund) in Cimerman and Langer, p. 22, pl. 12, figs. 1-3.

1993 Textularia pala (Czjzek) in Sgarrella and Moncharmont-Zei, p. 166, pl. 3, fig. 8.

2009 Textularia pala (Czjzek) in Milker et al., p. 215, pl. 1, figs. 13, 14.

2012 Textularia pala (Czjzek) in Milker and Schmiedl, p. 40, fig. 10.21-22.

**Textularia porrecta (Brady, 1884)**

1884 Textularia porrecta in Brady, p. 363, pl.43, fig. 4.

1970 Textularia Porrecta (Brady) in von Daniels, p. 70, pl. 2, fig. 4.

1991 Textularia Porrecta (Brady) in Cimerman and Langer, p. 22, pl. 11, figs. 1-4.

**Textularia truncata (Höglund, 1947)**

1947 Textularia truncata in Höglund, p. 175, pl. 12, figs. 8, 9.

1958 Textularia truncata (Höglung) in Le Calvez, p. 149, pl. 1, fig 5.

1991 Textularia truncata (Höglund) in Cimerman and Langer, p. 22, pl. 12, figs. 1-3.

**Textularia pseudorugosa (Lacroix, 1932)** Figure 6 (BB, CC)

1932 Textularia pseudorugosa in Lacroix, p. 19, figs. 19-22.

1958 Textularia pseudorugosa (Lacroix) in Le Calvez, p. 151.

1974 Textularia pseudorugosa (Lacroix) in Colom, p. 89, figs. 8 g-1.

1991 Textularia pseudorugosa (Lacroix) in Cimerman and Langer, p. 22, pl. 11, figs. 5-8.

Family VALVULINIDAE Berthelin. 1880

Subfamily VALVCLININAE Berthelin. 1880

Genus CLAVULINA d'Orbigny, 1826

**Clavulina difformis (Brady, 1884)** Figure 6 (E, F, G)

1884 Clavulina angularis d’Orbigny var. difformis in Brady, p. 392; pl. 48, figs 25-27.

1932 Clavulina difformis (Brady) in Parr, p. 5; pl. 1, fig. 6.

2009 Clavulina difformis (Brady) in Parker, p. 25; figs 19a-c.

2012 Clavulina difformis (Brady) in Debenay, p. 77.

**Clavulina multicamerata (Chapman, 1907)**

1884 Clavulina parisiensis (d'Orbigny) in Brady (part. non d'Orbigny, 1826) p. 395, pi. 48, figs. 17. 18 (not figs. 14-16).

1907 Clavulina multicamerata in Chapman, p. 127. pl. 9. fig. 5.

1937b Clavulina multicamerata (Chapman) in Cushman, p. 24. pl. 3, figs. 13-16.

1994 Clavulina multicamerata (Chapman) in Loeblich and Tappan, p. 33, pl. 47, figs. 11-15.

Family SPIRILLINIDAE Reuss and Fritsch, 1861

Genus MYCHOSTOMINA Berthelin, 1881

**Mychostomina revertens (Rhumbler, 1906)** Figure 6 (II, JJ)

1906 Spirillina vivipara (Ehrenberg) var. revertens in Rhumbler, p. 32, pI. 2, figs. 8-10.

1964 Spirillina revertens (Rhumbler) in Loeblich and Tappan, p. C600, pl. 475, fig, 3 (not fig. 4).

1974 Mychostomina revertens (Rhumbler) in Smith and Isham, p. 61. pl. I, figs. 1-3,7-9, pl. 2, figs. 1-5 (not figs. 6, 7, and not pI. 3, figs. 1-6), pl. 4, figs. 1-6.

1987 Mychostomina revertens (Rhumbler) in Loeblich and Tappan, p. 303, pl. 318, figs. 9-11,13-15 (not fig. 12).

1994 Mychostomina revertens (Rhumbler) in Loeblich and Tappan, p. 36, pl. 52, figs. 1-13.

Order SPIRILLINIDA Gorbachik and Mantsurova, 1980

Suborder SPIRILLININA Hohenegger and Piller, 1975

Family SPIRILLINIDAE Reuss and Fritsch, 1861

Genus SPIRILLINA Ehrenberg, 1843

**Spirillina vivipara (Ehrenberg, 1941)**

1841 Spirillina vivipara in Ehrenberg, p. 422, pl. 3, fig 41 (fide Ellis and Messina, 1940).

1930 Spirillina vivipara (Ehrenberg) in Heron-Allen and Earland, p. 178.

1958 Spirillina vivipara (Ehrenberg) in Le Calvez, p. 181.

1960 Spirillina vivipara (Ehrenberg) in Barker, pl. 85, figs. 1-5.

1974 Spirillina vivipara (Ehrenberg) in Colom, p. 139, figs. 23 c,d.

1987 Spirillina vivipara (Ehrenberg) in Loeblich and Tappan, p. 304, pl. 318, figs. 4-7.

1991 Spirillina vivipara (Ehrenberg) in Cimerman and Langer, p. 24, pl. 14, figs. 4-6.

Family PATELLINIDAE Rhumbler, 1906

Subfamily PATELLININAE Rhumbler, 1906

Genus PATELLINA Williamson, 1858

**Patellina corrugata (Williamson, 1858)** Figure 7 (C, D)

1858 Patellina corrugata in Williamson, p. 46, pl. 3, figs. 86-89.

1884 Patellina corrugata (Williamson) in Brady, p. 634, pl. 86, figs. 1-7.

1931 Patellina corrugata (Williamson) in Cushman, p. 11, pl. 2, figs. 6, 7.

1987 Patellina corrugata (Williamson) in Loeblich and Tappan, p. 84, pl. 320, figs. 7-14.

1991 Patellina corrugata (Williamson) in Cimerman and Langer, p. 24, pl. 14, figs. 7-12.

1994 Patellina corrugata (Williamson) in Jones, p. 93, pl. 86, figs. 1-7.

2003 Patellina corrugata (Williamson) in Murray, p. 24, fig. 9, no. 6, 7.

2012 Patellina corrugata (Williamson) in Milker and Schmiedl, fig. 11.21-23.

Order MILIOLIDA Lankester, 1885

Suborder MILIOLINA Delage and Herouard, 1896

Superfamily CORNUSPIRIDAE Schultze, 1854

Family CORNUSPIRIDAE Schultze, 1854

Subfamily CORNUSPIRINAE Schultze, 1854

Genus CORNUSPIRA Schultze, 1854

**Cornuspira involvens (Reuss, 1850)** Figure 7 (A, B)

1850 Operculina involvens in Reuss, p. 147, pl. 46, fig. 20 (fide Ellis and Messina, 1940).

1971 Cyclogira involvens (Reuss) in Murray, p. 53, pl. 18, figs. 1-3

1993 Cornuspira involvens (Reuss) in Sgarrella and Moncharmonz Zei, p. 167.

1992 Cornuspira involvens (Reuss) in Cimmermann and Langer, p. 25, pl. 15, figs. 4-7.

Family NUBECULARIIDAE Jones, 1875

Genus WIESNERELLA Cushman, 1933a

**Wiesnerella auriculata (Egger, 1893)** Figure 7 (G, H)

1893 Planispira auriculata in Egger, p.245-246, pl. 3, figs 13-15.

1915 Planispirina auriculata (Egger) in Heron-Allen and Earland, p. 590, pl. 46, figs. 3-7.

1929 Planispirina auriculata (Egger) in Cushman, p. 93, pl. 22, fig. 3.

1932 Planispirina auriculata (Egger) in Cushman, p. 72, pl. 16, fig. 6.

1987 Wiesnerella auriculata (Egger) in Loeblich and Tappan, p. 87, pl. 330, figs. 11-13.

1993 Wiesnerella auriculata (Egger) in Hottinger et al., p. 43, pl. 24, figs. 1-4.

2012 Wiesnerella auriculata (Egger) in Milker and Schmiedl, p. 46, fig. 12.4.

Superfamily MILIOLACEA Ehrenberg, 1839

Family SPIROLOCULINIDAE Wiesner, 1920

Subfamily SPIROLOCULININAE Wiesner, 1920

Genus ADELOSINA d’Orbigny, 1826

**Adelonsina carinata-striata (Wiesner, 1923)** Figure 7 (L, M, N)

1923 Adelosina milletti var. carinata-striata in Wiesner, p. 77, pl. 14, figs. 190, 191.

1970 Quinqueloculina milletti var. carinata-striata (Wiesner) in von Daniels, p. 74, pl. 2, figs. 17 a-c, textfig. 49.

1991 Adelosina carinata-striata (Wiesner) in Cimerman and Langer, p. 28, pl. 20, figs. 1-4.

**Adelosina cliarensis (Heron-Allen and Earland, 1930)** Figure 7 (O, P, Q)

1930 Quinqueloculina cliarensis in Heron-Allen and Earland, p. 58, pl. 3, figs. 26, 31.

1958 Quinqueloculina cliarensis (Heron-Allen and Earland) in Le Calvez and Le Calvez, p. 186, pl. 5, figs. 40, 41.

1958 Quinqueloculina cliarensis (Heron-Allen and Earland) in Le Calvez and Le Calvez, p. 157, pl. 1, figs. 10, 11.

1991 Adelosina cliarensis (Heron-Allen and Earland) in Cimerman and Langer, p. 26, pl. 18, figs. 1-4.

**Adelosina dubia (d’Orbigny, 1826)**

1826 Triloculina dubia in d’Orbigny, p. 300, no. 24.

1923 Adelosina dubia (d’Orbigny) in Wiesner, p. 77, pl. 14, fig. 193.

1958 Triloculina dubia (d’Orbigny) in Le Calvez and Le Calvez., p. 197, pl. 14, figs. 164-166.

1991 Adelosina dubia (d’Orbigny) in Cimerman and Langer, p. 27, pl. 18, figs. 5-7.

**Adelosina elegans (Williamson, 1858)**

1858 Miliolina bicornis (Walker and Jacob) var. elegans in Williamson, p. 88, pl. 7, fig. 195.

1923 Adelosina elegans (Williamson) in Wiesner, p. 80, pl. 15, fig. 209.

1958 Quinqueloculina williamsoni in Le Calvez and Le Calvez, p. 177, pl. 5, fig. 45.

1991 Adelosina elegans Williamson in Cimerman and Langer, p. 27, pl. 20, figs. 5-6.

**Adelosina laevigata (d’Orbigny, 1826)** Figure 7 (R, S)

1826 Adelosina laevigata in d’Orbigny: p. 304, no.1.

1846 Adelosina laevigata (d’Orbigny) in d’Orbigny,p. 302, pl. 20, figs. 22-24.

1987 Adelosina laevigata (d’Orbigny) in Loeblich and Tappan, p. 90, pl. 337, figs. 5-12.

2012 Adelosina laevigata (d’Orbigny) in Milker and Schmiedl, p. 48, fig. 12.18-19.

**Adelosina italica (Terquem 1878)**

1878 Quinqueloculina italica in Terquem, p. 69, pl. 7, figs. 17a-20c.

1993 Adelosina italica in Sgarrella and Moncharmont-Zei, p. 179, pl. 7, figs. 13-14.

Genus SPIROLOCULINA d’Orbigny, 1826

**Spiroloculina ornata (d’Orbigny, 1839)**

1839 Spiroloculina ornata in d’Orbigny, p. 167, pl. 12, fig. 7.

1958 Spiroloculina ornata (d’Orbigny) in Le Calvez and Le Calvez, p. 207, pl. 8, fig. 83.

1977 Spiroloculina ornata (d’Orbigny) in Le Calvez, p. 94, pl. 18, figs. 1-4.

1991 Spiroloculina ornata (d’Orbigny) in Cimerman and Langer, p. 30, pl. 23, figs. 8-11.

**Spiroloculina ornata (d’Orbigny) var. tricarinata (Le Calvez and Le Calvez, 1958)**

1958 Spiroloculina ornata (d’Orbigny) var. tricarinata in Le Calvez and Le Calvez, p. 207, pl. 8, figs. 84, 85.

1991 Spiroloculina ornata (d’Orbigny) var. tricarinata in Cimerman and Langer, p. 30, pl. 23, figs. 4-7.

Family HAUERINIDAE Schwager, 1876

Subfamily SIPHONAPERTINAE Saidova, 1975

Genus SIPHONAPERTA Vella, 1957

**Siphonaperta agglutinans (d’Orbigny, 1839)**

1839a Quinqueloculina agglutinans in d’Orbigny, p. 195, pl. 12, figs. 11, 12.

1958 Quinqueloculina agglutinans (d’Orbigny) in Le Calvez and Le Calvez, p. 166, pl. 9, figs. 103, 104.

1977 Quinqueloculina agglutinans (d’Orbigny) in Le Calvez, p. 54, pl. 7, figs. 1-4.

1991 Siphonaperta agglutinans (d’Orbigny) in Cimerman and Langer, p. 31, pl. 25, figs. 1-3.

**Siphonaperta aspera (d’Orbigny, 1826)**

1826 Quinqueloculina aspera in d’Orbigny, p. 301, no. 11.

1958 Quinqueloculina aspera (d’Orbigny) in Le Calvez and Le Calvez, p. 168, pl. 9, figs. 101, 102.

1991 Siphonaperta aspera (d’Orbigny) in Cimerman and Langer, p. 31, pl. 25, figs. 4-6.

Subfamily HAUERININAE Schwager, 1876

Genus CYCLOFORINA Luczowksa, 1972

**Cycloforina contorta (d’Orbigny, 1846)** Figure 7 (V, W, X)

1846 Quinqueloculina contorta in d’Orbigny, p. 298, pl. 20, figs. 4-6.

1923 Quinqueloculina contorta (d’Orbigny) in Wiesner, p. 46, pl. 6, fig. 56.

1958 Quinqueloculina contorta (d’Orbigny) in Le Calvez and Le Calvez, p. 171, pl. 12, figs. 140-142.

1987 Cycloforina contorta (d’Orbigny) in Loeblich and Tappan, p. 33, pl. 342, figs. 4-9.

1991 Cycloforina contorta (d’Orbigny, 1846) in Cimerman and Langer, p. 32, pl. 27, figs. 7-11.

Genus LACHLANELLA Vella, 1957

**Lachlanella variolata (d’Orbigny, 1826)** Figure 7 (Y, Z, AA)

1826 Quinqueloculina variolata in d’Orbigny, p. 24.

1929 Triloculina carinata (d’Orbigny) in Cushman, p. 65, pl. 17, fig. 5.

1991 Lachlanella variolata (d’Orbigny) in Cimerman and Langer, p. 35, pl. 31, figs. 1-12.

1993 Quinqueloculina variolata (d’Orbigny) in Sgarrella and Moncharmont Zei, p. 175, pl. 8, fig.1.

Genus QUINQUELOCULINA d’Orbigny, 1826

**Quinqueloculina berthelotiana (d’Orbigny, 1839b)**

1839b Quinqueloculina berthelotiana in d’Orbigny, p. 142, pl. 3, fig. 25-27.

1923 Quinqueloculina berthelotiana (d’Orbigny) in Wiesner, p. 48, pl. 6, fig. 67.

1958 Quinqueloculina berthelotiana (d’Orbigny) in Le Calvez and Le Calvez, p. 173, pl. 10, figs. 115-117.

1974 Quinqueloculina berthelotiana (d’Orbigny) in Colom, p. 188, figs. 59 e-g.

1991 Quinqueloculina berthelotiana (d’Orbigny) in Cimerman and Langer, p. 36, pl. 32, figs. 5-7.

**Quinqueloculina bidentata (d’Orbigny, 1839)** Figure 7 (DD, EE)

1839b Quinqueloculina bidentata in d’Orbigny, p. 197, pl. 12, figs. 18-20.

1929 Quinqueloculina bidentata (d’Orbigny) in Cushman, p. 22, pl. 1, fig. 2.

1977 Quinqueloculina bidentata (d’Orbigny) in Le Calvez, p. 64, 65, figs. 1-2.

1991 Quinqueloculina bidentata (d’Orbigny) in Cimerman and Langer, p. 36, pl. 32, figs. 10-14.

**Quinqueloculina bosciana (d’Orbigny, 1839)** Figure 7 (FF, GG)

1839a Quinqueloculina bosciana in d’Orbigny, p. 191, pl. 11, figs. 22-24.

1977 Quinqueloculina bosciana (d’Orbigny) in Le Calvez, p. 66, pl. 10, figs. 1-3.

1991 Quinqueloculina bosciana (d’Orbigny) in Cimerman and Langer, p. 36, pl. 33, figs. 5-7.

**Quinqueloculina disparilis (d’Orbigny, 1826)**

1826 Quinqueloculina disparilis in d’Orbigny, p. 302, no 21.

1893 Quinqueloculina disparilis (d’Orbigny) in Schlumberger, p. 212, pl. 2, figs. 55-57.

1923 Quinqueloculina disparilis (d’Orbigny) in Wiesner, p. 47, pl. 6, figs. 60, 61.

1958 Quinqueloculina disparilis (d’Orbigny) in Le Calvez and Le Calvez, p. 180, pl. 4, figs. 26, 27.

1974 Quinqueloculina disparilis (d’Orbigny) in Colom, p. 200, figs. 55 a-g.

1991 Quinqueloculina disparilis (d’Orbigny) in Cimerman and Langer, p. 36, pl. 33, fugs. 1-4.

**Quinqueloculina jugosa (Cushman, 1944)** Figure 7 (HH, JJ)

1878 Quinqueloculina costata in Terquem, p. 63, pl. 6, figs. 3, 5.

1944 Quinqueloculina seminulum (Linnaeus) var. jugosa in Cushman, p. 13, pl. 2, fig. 15.

1958 Quinqueloculina seminulum (Linnaeus) var. jugosa Cushman in Le Clavez, p. 178, pl. 4, figs. 20, 21.

1991 Quinqueloculina jugosa (Cushman) in Cimerman and Langer, p. 37, pl. 33, figs. 12-14.

**Quinqueloculina laevigata (d’Orbigny, 1839)** Figure 8 (A, B, C)

1839b Quinqueloculina laevigata in d’Orbigny, p. 143, pl. 3, figs. 32-33.

1923 Miliolina laevigata (d’Orbigny) in Wiesner, p. 55, pl. 8, figs. 94-96.

1929 Quinqueloculina laevigata (d’Orbigny) in Cushman, p. 30, pl. 4, fig. 3.

1958 Quinqueloculina laevigata (d’Orbigny) in Le Calvez and Le Calvez, p. 184, pl. 10, figs. 112-114.

1991 Quinqueloculina laevigata (d’Orbigny) in Cimerman and Langer, p. 37, pl. 33, figs. 8-11.

**Quinqueloculina lata (Terquem, 1876)**

1876 Quinqueloculina lata in Terquem: p. 82, pl. 11, fig. 8a-c (fide Ellis and Messina, 1940ff).

1958 Quinqueloculina lata (Terquem) in Le Calvez, p. 158, pl. 2, figs. 26-28.

1993 Quinqueloculina lata (Terquem) in Sgarrella and Moncharmont-Zei, p. 172, pl. 5, fig. 15.

2003 Quinqueloculina lata (Terquem) in Murray, p. 17, fig. 4, no. 9, 10.

2012 Quinqueloculina lata (Terquem) in Milker and Schmiedl, p. 58, fig. 15.16.

**Quinqueloculina parvula (Schlumberger, 1893)**

1893 Quinqueloculina parvula in Schlumberger, p. 255, pl. 3, figs. 8, 9.

1958 Quinqueloculina parvula (Schlumberger) in Le Calvez and Le Calvez, p. 184, pl. 10, figs. 131-133.

1991 Quinqueloculina parvula (Schlumberger) in Cimerman and Langer, p. 37, pl. 34, figs 6-8.

**Quinqueloculina patagonica (d’Orbigny, 1839)**

1839a Quinqueloculina patagonica in d’Orbigny, p. 74, pl. 4, figs. 14-16.

1949 Quinqueloculina laevigata (d’Orbigny) in Said, p. 10, pl. 1, fig. 27.

1993 Quinqueloculina patagonica (d’Orbigny) in Hottinger et al., p. 60, pl. 55, figs. 11-17.

**Quinqueloculina pseudobuchiana (Luczkowska, 1974)** Figure 8 (D, F)

1974 Quinqueloculina pseudobuchiana in Luczkowska, p. 58, pl. 4, fig. 5; pl. 5, figs. 1, 2.

1991 Quinqueloculina pseudobuchiana (Luczkowska) in Cimerman and Langer, p. 38, pl. 35, figs. 1-4.

2012 Quinqueloculina pseudobuchiana (Luczkowska) in Milker and Schmiedl, figs. 15.28-29.

**Quinqueloculina seminula (Linnaeus, 1758)** Figure 8 (G, H)

1758 Quinqueloculina seminula in Linnaeus, p. 786, pl. 2, figs 1 a-c (fide Ellis and Messina, 1940).

1893 Quinqueloculina seminulum (Linnaeus) in Schlumberger, p. 208, pl. 4, figs. 80, 81.

1923 Miliolina seminulum (Linnaeus) in Wiesner, p. 66, pl. 12, fig. 148.

1960 Quinqueloculina seminulum (Linnaeus) in Barker, pl. 5, fig. 6.

1970 Quinqueloculina seminulum (Linnaeus) in von Daniels, p. 75, pl. 3, fig. 4.

1991 Quinqueloculina seminula (Linnaeus) in Cimerman and Langer, p. 38, pl. 34, figs. 9-12.

**Quinqueloculina stelligera (Schlumberger, 1893)**

1893 Quinqueloculina stelligera in Schlumberger, p. 210, pl. 2, figs. 58, 59.

1923 Miliolina schlumbergeri in Wiesner, p. 49, pl. 6, fig. 73.

1958 Quinqueloculina stelligera (Schlumberger) in Le Calvez and Le Calvez, p. 174, pl. 11, figs. 125, 126.

1991 Quinqueloculina stelligera (Schlumberger) in Cimerman and Langer, p. 38, pl. 34, figs. 13-15.

**Quinqueloculina viennensis (Le Calvez and Le Calvez, 1958)**

1958 Quinqueloculina viennensis (Le Calvez and Le Calvez) in Le Calvez and Le Calvez, p. 187, pl. 5, figs. 42, 44, 45.

1993 Quinqueloculina viennensis (Le Calvez and Le Calvez) in Sgarrella and Moncharmont Zei, p. 176, pl. 7, fig. 8.

2005 Quinqueloculina viennensis (Le Calvez and Le Calvez) in Rasmussen, p. 64, pl. 4, fig. 13.

2012 Quinqueloculina viennensis (Le Calvez and Le Calvez) in Milker and Schmiedl, p. 60, fig. 16.5-7.

Subfamily MILIOLINELLINAE Vella, 1957

Genus AFFINETRINA Luczkowska, 1972

**Affinetrina gualtieriana (d’Orbigny, 1839a)** Figure 7 (T, U)

1839a Triloculina gualtieriana in d’Orbigny, p. 170, pl. 9, figs. 5-7.

1974 Affinetrina gualteriana (d’Orbigny) in Luczkowska, p. 109, pl. 26, figs. 1, 2; text-fig. 38.3.

2012 Affinetrina gualtieriana (d’Orbigny) in Milker and Schmiedl, p. 61, Fig. 16.9-10.

Genus MILIOLINELLA Wiesner, 1931

**Miliolinella elongata (Kruit, 1955)**

1955 Miliolinella circularis (Bornemann) var. elongata in Kruit, p. 110, pl. 1, fig. 15.

1991 Miliolinella elongata (Kruit) in Cimerman and Langer, p. 41, pl. 37, fig. 8.

1993 Miliolinella circularis elongata (Kruit) in Sgarrella and Moncharmont-Zei, p. 187, pl. 10, fig. 5.

2012 Miliolinella elongata (Kruit) in Milker and Schmiedl, fig. 16.23-24.

**Miliolinella labiosa (d’Orbigny, 1839)**

1839a, Triloculina labiosa in d’Orbigny, p. 178, pl. 10, figs. 12-14.

1923 Miliolina labiosa (d’Orbigny) in Wiesner, p. 71, pl. 134, fig. 171.

1929 Triloculina labiosa (d’Orbigny) in Cushman, p. 60, pl. 15, fig. 3.

1958 Triloculina labiosa (d’Orbigny) in Le Calvez and Le Calvez, p. 196, pl. 14, figs, 168, 169.

1991 Miliolinella labiosa (d’Orbigny) in Cimerman and Langer, p. 41, pl. 38, figs. 1-3.

**Miliolinella subrotunda (Montagu, 1803)** Figure 8 (I, J)

1803 Vermiculum subrotundum in Montagu, p. 521, (fide Ellis and Messina, 1940).

1923 Miliolina subrotunda (Walker and Boys) in Wiesner, p. 69, pl. 13, figs. 165-169.

1970 Miliolinella subrotunda (Walker and Boys) in von Daniels, p. 77, pl. 4, fig. 2, textfig. 52.

**Miliolinella webbiana (d’Orbigny, 1839)** Figure 8 (K, L, M)

1839 Triloculina webbiana in d’Orbigny, p. 140, pl.3, figs. 13-15.

1958 Triloculina webbiana (d’Orbigny) in Le Calvez and Le Calvez, p. 195, pl. 15, figs. 176-178.

1991 Miliolinella webbiana (d’Orbigny) in Cimerman and Langer, p. 42, pl. 39, figs. 1-3.

1993 Miliolinella webbiana (d’Orbigny) in Sgarrella and Moncharmont-Zei, p. 187, pl. 10, fig. 6.

2009 Miliolinella webbiana (d’Orbigny) in Milker et al., p. 215, pl. 1, fig. 19.

2012 Miliolinella webbiana (d’Orbigny) in Milker and Schmiedl, p.65, fig. 17.1, 17.2.

Genus PSEUDOTRILOCULINA Cherif, 1970

**Pseudotriloculina laevigata (d’Orbigny, 1826)** Figure 7 (BB, CC)

1826 Triloculina laevigata in d’Orbigny, p. 300, no. 15.

1923 Miliolina laevigata (d’Orbigny) in Wiesner, p. 55, pl. 8, figs. 94-96.

1958 Triloculina laevigata (d’Orbigny) in Le Calvez and Le Calvez, p. 19, pl. 6, figs. 62-64.

1991 Pseudotriloculina laevigata (d’Orbigny) in Cimerman and Langer, p. 43, pl. 39, figs. 8-12.

**Pseudotriloculina oblonga (Montagu, 1803)**

1803 Vermiculum oblongum in Montagu, p. 522, pl. 14, fig. 9 (fide Ellis and Messina, 1940).

1839b Triloculina oblonga (Montagu) in d’Orbigny, p. 175, pl. 10, figs. 3, 5.

1958 Triloculina oblonga (Montagu) in Le Calvez and Le Calvez, p. pl. 6, figs. 60,61.

1958 Triloculina oblonga (Montagu) in Le Calvez, p. 115, figs. 1-2.

1991 Pseudotriloculina oblonga (Montagu) in Cimerman and Langer, p. 43, pl. 40, figs. 1-4.

Genus TRILOCULINA d’Orbigny, 1826

**Triloculina affinis (d’Orbigny, 1852)**

1852 Triloculina affinis in d’Orbigny, p. 161.

1905 Triloculina affinis (d’Orbigny) in Fornasini, pl. 1, fig. 1.

1959 Triloculina affinis (d’Orbigny) in Graham and Militante, p. 52, pl. 7, figs, 5, 6.

1993 Triloculina affinis (d’Orbigny) in Hottinger et al., p. 64, pl. 65, figs. 7-10, pl. 66, figs. 1-3.

**Triloculina marioni (Schlumberger, 1893)**

1893 Triloculina marioni in Schlumberger, p.204, pl. 1, figs. 38-41.

1958 Triloculina marioni (Schlumberger) in Le Calvez and Le Calvez, p. 191, pl. 6, figs. 54-56.

1991 Triloculina marioni (Schlumberger) in Cimerman and Langer, p. 46, pl. 43, figs. 1-5.

**Triloculina plicata (Terquem, 1878)**

1878 Triloculina plicata in Terquem, p. 61, pl. 6, figs. 2 a, b.

1923 Triloculina plicata (Terquem) in Wiesmer, p. 62, pl. 11, figs. 129, 130.

1958 Triloculina plicata (Terquem) in Le Calvez and Le Calvez, p. 189, pl. 14, figs. 162, 163.

1991 Triloculina plicata (Terquem) in Cimerman and Langer, p. 46, pl. 43, figs. 8-10.

Subfamily SIGMOILINITINAE Luczkowa, 1974

Genus SIGMOILINITA Seiglie, 1965

**Sigmoilinita costata (Schlumberger, 1893)**

1893 Sigmoilina costata in Schlumberger, p. 203, pl. 1, figs. 51, 52.

1958 Sigmoilina costata (Schlumberger) in Le Calvez and Le Calvez, p. 20, pl. 7, figs. 69, 70.

1991 Sigmoilinita costata (Schlumberger) in Cimerman and Langer, p. 47, pl. 45, figs. 1-6.

Family TUBINELLIDAE Rhumbler, 1906

Genus articulina d’Orbigny, 1826

**Articulina carinata (Wiesner, 1923)** Figure 7 (I, J, K)

1923 Articulina sagra d’Orbigny var. carinata in Wiesner, p. 74, pl. 19, fig. 188.

1970 Articulina pacifica (Cushman) in von Daniels, p. 78, textfig. 53.

1991 Articulina carinata (Wiesner) in Cimerman and Langer, p. 48, pl. 47, figs. 1-5.

Superfamily SORITACEA Ehrenberg, 1839

Family PENEROPLIDAE Schultze, 1854

Genus LAEVIPENEROPLIS Šulc, 1936

**Laevipeneroplis karreri (Wiesner, 1923)** Figure 8 (P, Q)

1923 Peneroplis karreri in Wiesner, p. 96, pl. 20, fig. 285.

1991 Laevipeneroplis karreri (Wiesner) in Cimerman and Langer, p. 49, pl. 48, figs. 1-7.

Genus PENEROPLIS de Monfort, 1808

**Peneroplis pertusus (Forskål, 1775)** Figure 8 (U, V, W)

1775 Nautilus pertusus in Forskål, p. 125 (fide Ellis and Messina, 1940).

1917 Peneroplis pertusus (Forskål) in Cushman, p. 86, pl. 36, fig. 1, pl. 37, figs. 1, 2, 6.

1974 Peneroplis pertusus (Forskål) in Colom, p. 219, fig. 64 j.

1991 Peneroplis pertusus (Forskål) in Cimerman and Langer, p. 49, pl. 49, figs. 1-8.

**Peneroplis planatus (Fichtel and Moll, 1798)** Figure 8 (R, S, T)

1798 Nautilus planatus in Fichtel and Moll, p. 91, pl. 16, figs. a-h.

1826 Peneroplis planatus (Fichtel and Moll) in d’Orbigny, p. 285, no. 1.

1858 Peneroplis planatus (Fichtel and Moll) in Williamson, p. 45, pl. 3, figs. 84, 85.

1960 Peneroplis planatus (Fichtel and Moll) in Barker, pl. 13, fig. 15.

1974 Peneroplis planatus (Fichtel and Moll) in Colom, p. 219, figs. 64, h-i, k.

1987 Peneroplis planatus (Fichtel and Moll) in Baccaert, p. 58, pl. 17, figs. 1-4, pl. 18, fig. 1.

1987 Peneroplis planatus (Fichtel and Moll) in Loeblich and Tappan, p. 371, pl. 391, figs. 7, 8.

1991 Peneroplis planatus (Fichtel and Moll) in Cimerman and Langer, p. 50, pl. 50., figs. 1-6.

Family SORITIDAE Ehrenberg, 1839

Subfamily SORITINAE Ehrenberg, 1839

Genus SORITES Ehrenberg, 1839

**Sorites orbiculus (Ehrenberg, 1839)** Figure 8 (X, Y)

1775 Nautilus orbiculus in Forskål, p. 125 (fide Ellis and Messina, 1940).

1839 Sorites orbiculus in Ehrenberg, p. 134.

1852 Orbiculina complanata in Williamson, p. 115.

1961 Sorites orbiculus (Ehrenberg) in Lehmann, p. 641, pl. 8, figs. 1-8.

1977 Sorites orbiculus (Ehrenberg) in Hottinger, p. 94, figs. 9 B. 30D, E, 32 B.

1987 Sorites orbiculus (Ehrenberg) in Baccaert, pl. 28, figs. 1, 2, pl. 29, figs. 1 a, b.

1987 Sorites orbiculus (Ehrenberg) in Loeblich and Tappan, p. 382, pl. 419, figs. 4-10.

1991 Sorites orbiculus (Ehrenberg) in Cimerman and Langer, p. 50, pl. 51, figs. 1-5.

Family VAGINULIDAE Reuss, 1862

Subfamily LENTICULINIDAE Chapman, Parr, and Collins, 1934

Genus LENTICULINA Lamarck, 1804

**Lenticulina cultrata (de Montfort, 1808)** Figure 8 (DD, EE, FF)

1808 Robulus cultratus in de Montfort, p. 214, pl. 25, no. 14,15 (fide Ellis and Messina, 1944).

1839b Robulina cultrata (Montfort) in d’Orbigny, p. 26, pl. 5, figs. 19, 20.

1974 Lenticulina cultrata (Montfort) in Le Calvez, p. 58, textfig. 1.

1991 Lenticulina cultrata (Montfort) in Cimerman and Langer, p. 51, pl. 53, figs. 5-6.

**Lenticulina gibba (d’Orbigny, 1826)**

1826 Cristellaria gibba in d’Orbigny, p. 292, no. 17.

1839a Cristellaria gibba in d’Orbigny, p. 40, pl. 7, figs. 20, 21.

1913 Cristellaria gibba (d’Orbigny) in Cushman, p. 105, pl. 25, fig. 4.

1974 Robulus gibbus (d’Orbigny) in Colom, p. 96, fig. 11g.

1977b Lenticulina gibba (d’Orbigny) in Le Calvez, p. 25, fig. 1.

Order LAGENIDA Lankester, 1885

Superfamily NODOSARIACEA Ehrenberg, 1838

Family LAGENIDAE Reuss, 1862

Genus LAGENA Walker and Jacob, 1798

**Lagena striata (d’Orbigny, 1839)** Figure 8 (BB, CC)

1839b Oolina striata in d’Orbigny, pl. 5, fig. 12.

1923 Lagena substriata (Williamson) in Cushman, p. 56, pl. 10, fig. 11.

1970 Lagena striata (d’Orbigny) in von Daniels, p. 79, pl. 4, fig. 9°.

1991 Lagena striata (d’Orbigny) in Cimerman and Langer, p. 53, pl. 55, figs. 6-7.

Family ELLIPSOLAGENIDAE Silvestri, 1923

Subfamily ELLIPSOLAGENINAE Silvestri, 1923

Genus FISSURINA Reuss, 1850

**Fissurina lucida (Williamson, 1858)**

1858 Entosolenia marginata var. lucida in Williamson, p. 10, pl. 1, figs. 22, 23.

1991 Fissurina lucida (Williamson) in Cimerman and Langer, p. 55, pl. 59, fig. 1.

Order BULIMINIDA Fursenko, 1958

Superfamily CASSIDULINACEA d'Orbigny, 1839

Family CASSIDULINIDAE d’Orbigny, 1839

Subfamily CASSIDULININAE d'Orbigny, 1839

Genus Cassidulina d’Orbigny, 1826

**Cassidulina obtusa (Williamson, 1858)** Figure 10 (C, D)

1858 Cassidulina obtusa in Williamson, p. 69, pl. 6, figs. 143, 144.

1884 Cassidulina crassa (d’Orbigny) in Brady, p. 429, pl. 54, figs. 4, 5.

1911 Cassidulina crassa (d’Orbigny) in Cushman, p. 97, text-fig. 151.

1945 Cassidulina crassa (d’Orbigny) in Cushman, p. 288, figs. 18, 19.

1958 Cassidulina crassa (d’Orbigny) in Parker, p. 271, pl. 4, fig. 12.

1987 Cassidulina crassa (d’Orbigny) in Jorissen, p. 41, pl. 1, fig. 3.

1991 Cassidulina crassa (d’Orbigny) in Rasmussen, p. 364, fig. 7, no. 2.

1994 Cassidulina crassa (d’Orbigny) in Jones, p. 60, pl. 54, figs. 4, 5 [cop. Brady 1884, figs. 4, 5].

2003 Cassidulina obtusa (Williamson) in Murray, p. 21, fig. 6, no. 11, 12.

2005 Cassidulina obtusa (Williamson) in Rasmussen, p. 82, pl. 10, figs. 2-4.

2008 Cassidulina crassa (d’Orbigny) in Abu-Zied et al., p. 52, pl. 2, figs. 3, 4.

2009 Cassidulina crassa (d’Orbigny) in Milker et al., p. 216, pl. 2, fig. 17.

2012 Cassidulina obtusa (Williamson) in Milker and Schmiedl, p. 84, fig. 20.7-9.

Family BULIMINIDAE Jones, 1875

Genus Bulimina d’Orbigny, 1826

**Bulimina gibba (Fornasini, 1901)**

1884 Bulimina elegans d’Orbigny in Brady, p. 398, pl. 50, figs. 1, 2.

1901 Bulimina gibba (Fornasini) in Fornasini, p. 378, figs. 32, 34.

1958 Bulimina gibba (Fornasini) in Parker, p. 261, pl. 2, figs. 21, 22.

1960 Bulimina gibba (Fornasini) in Hofker, p. 248, pl. D, figs. 91-95.

1994 Bulimina gibba (Fornasini) in Jones, p. 54, pl. 50, figs. 1, 2.

2005 Bulimina gibba (Fornasini) in Rasmussen, p. 86, pl. 11, fig. 5.

2012 Bulimina gibba (Fornasini) in Milker and Schmiedl, fig. 20.22.

Genus PROTOGLOBOBULIMINA Hofker, 1951

**Protoglobobulimina pupoides (d'Orbigny, 1846)**

1846 Bulimina pupoides in d’Orbigny, p. 185, pl. II, figs. 11, 12.

1884 Bulimina pupoides d’Orbigny in Brady, p. 400, pl. 50, fig. 15.

1947 Bulimina pupoides d’Orbigny in Cushman and F.L. Parker, p. 105, pl. 25, figs. 3-7.

1944 Bulimina pupoides (d'Orbigny) elongate var. in Leroy, p. 26, pl. I, fig. 4.

1951 Protoglobobulimina pupoides (d'Orbigny) in Hofker, p. 252, text fig. 1 68.

1987 Protoglobobulimina pupoides (d'Orbigny) in Loeblich and Tappan, p. 522, pl. 572, figs. 1-6.

1994 Protoglobobulimina pupoides (d'Orbigny) in Loeblich and Tappan, p. 125, pl. 244, figs. 8-10.

Order ROTALIIDA Lankester, 1885

Superfamily DISCORBACEA Ehrenberg, 1838

Family BAGGINIDAE Cushman, 1927

Genus VALVULINERIA Cushman, 1926

**Valvulineria minuta (Parker, 1954)** Figure 9 (X, Y)

1954 Valvulineria minuta Parker in Parker, p. 527, pl. 9, figs. 4-6.

1993 Valvulineria minuta (Parker) in Sgarrella and Moncharmont-Zei, p. 220, pl. 18, figs. 3, 4.

2012 Valvulineria minuta (Parker) in Milker and Schmiedl, p. 91, figs. 21.18-19.

Family DISCORBIDAE Ehrenberg, 1838

Genus DISCONORBIS Sellier de Civrieux, 1977

**Disconorbis bulbosus (Parker, 1954)**

1954 Discorbis bulbosus in Parker, p. 523, pl. 8, figs. 10-12 (fide Ellis and Messina, 1940).

1987 Disconorbis bulbosus (Parker) in Loeblich and Tappan, p. 557, pl. 602, figs. 10-15.

1991 Disconorbis bulbosus (Parker) in Cimerman and Langer, p. 66, pl. 70, figs. 1-2.

Genus NEOEPONIDES Reiss, 1960

**Neoeponides bradyi (Le Calvez, 1974)**

1884 Pulvinulina berthelotiana (d'Orbigny) in Brady (non Rotalina berthelotiana d'Orbigny, 1839b), p. 701, pl. 106, fig. 1.

1960 Eponides berthelotianus (d'Orbigny) in Barker, p. 218, pl. 106, fig. L.

1966 Neoeponides berthelotianus (d'Orbigny) in Belford, p. 117, pl. 17, figs. 1-6.

1975 Neoeponides berthelotianus (d'Orbigny) in Saidova, pl. 63, fig. 3.

1974 Neoeponides bradyi in Le Calvez, p. 64.

1984 Neoeponides berthelotianus (d'Orbigny) in Rogl and Hansen, pl. 7. figs. 1-6.

1993 Neoeponides berthelotianus (d'Orbigny) in Hottinger et al., p. 337, pl. I, figs. 5-8.

1994 Neoeponides bradyi (Le Calvez) in Loeblich and Tappan, p. 138, pl. 279, figs. 1-9.

Family ROSALINIDAE Reiss, 1963

Genus GAVELINOPSIS Hofker, 1951

**Gavelinopsis praegeri (Heron-Allen and Earland, 1913)** Figure 10 (I, J)

1913 Discorbina praegeri in Heron-Allen and Earland, p. 122, pl. 10, figs. 8-10 (fide Ellis and Messina, 1940).

1972 Gavelinopsis praegeri (Heron-Allen and Earland) in Rosset-Moulinier, p. 167, pl. 9, figs. 27, 28.

1987 Gavelinopsis praegeri (Heron-Allen and Earland) in Loeblich and Tappan, p. 560, pl. 608, figs. 6-12.

1991 Gavelinopsis praegeri (Heron-Allen and Earland) in Cimerman and Langer, p. 66, pl. 70, figs. 3-4.

Genus NEOCONORBINA Hofker, 1951

**Neoconorbina terquemi (Rzehak, 1888)** Figure 10 (K, L)

1876 Rosalina orbicularis in Terquem, p. 75, pl. 9, figs. 4 a, b (fide Ellis and Messina, 1940).

1888 Discorbina terquemi in Rzehak, p. 228.

1958 Discorbis orbicularis (Terquem) in Le Calvez, p. 183.

1970 Neoconorbina terquemi (Rzehak) in von Daniels, p. 186, pl. 9, figs. 29, 30.

1974 Discorbis orbicularis (Terquem) in Colom, p. 125, fig. 21 k.

1987 Neoconorbina terquemi (Rzehak) in Loeblich and Tappan, p. 560, pl. 609, figs. 8-10.

1991 Neoconorbina terquemi (Rzehak) in Cimerman and Langer, p. 66, pl. 70, figs. 5-7.

**Neoconorbina concinna (Brady, 1884)** Figure 9 (NN, OO, PP)

1884 Discorbina concinna in Brady, p. 646. pI. 90. figs. 7. 8.

1934 Trelomphalus concinnus (Brady) in Cushman, p. 96, pl. 11, figs. 8, 9, pl. 12, figs. 13-15.

1957 Trelomphalus concinnus (Brady) in Todd, p. 292, pl. 92, fig. 4.

1965 Trelomphalus concinnus (Brady) in Todd, 1965, p. 39.

1952 Rosalina concinna (Brady) in Bermudez, p. 34.

1965 Rosalina concinna (Brady) in Todd, p. 10, pl. 4, fig. 3.

1976 Rosalina globularis d'Orbigny in Sellier de Civrieux (non R. globularis d'Orbigny, 1826), p. 181, pl. 1, figs. 1-9, pl. 2, figs. 1-8, pl. 3, figs. 1-9, pl. 4, figs, 1-9.

1985 Neoconorbina (Treromphaloides) concinnus (Brady) in Banner et al., p. 166, pl. 1, figs, 6-10, pl. 2. figs. 1-3.

1987 Tretomphaloides concinnus (Brady) in Loeblich and Tappan, p. 562, pl. 613, figs. 1-6.

1994 Tretomphalus concinnus (Brady) in Loeblich and Tappan, p. 140, pl. 288, figs. 1-10.

Genus ROSALINA d’Orbigny, 1826

**Rosalina bradyi (Cushman, 1915)** Figure 9 (DD, EE)

1884 Discorbina globularis; in Brady (not d'Orbigny), p. 178, pl. 86, figs. 8a-c.

1915 Discorbis globularis; (d'Orbigny) var. bradyi in Cushman, p.12.

1951 Discopulvinulina bradyi (Cushman) in Hofker, p. 452, figs. 310 a, b.

1954 Rosalina bradyi (Cushman) in Hornibrook and Vella, p. 26.

1991 Rosalina bradyi (Cushman) in Cimerman and Langer, p. 66, pl. 71, figs. 1-5.

**Rosalina bulloides (d'Orbigny, 1839)** Figure 10 (A, B)

1839a Rosalina bulloides in d'Orbigny, p. 98, pl. 3, figs. 2-5.

1985 Rosalina (Tretomphalus) bulloides (d'Orbigny) in Banner et al., p. 164, pl. 1, figs. 1-5.

1987 Tretomphalus bulloides (d'Orbigny) in Loeblich and Tappan, p. 262, pI. 612, figs. 1-11.

1991 Tretomphalus bulloides (d'Orbigny) in Cimerman and Langer, p. 67, pl. 72, figs. 3-5.

**Rosalina globularis (d’Orbigny, 1826)** Figure 9 (FF, GG)

1826 Rosalina globularis in d’Orbigny, p. 271, pl. 13, figs. 1-4.

1987 Rosalina globularis d’Orbigny in Loeblich and Tappan, p. 161, pl. 610, figs. 1-5; pl. 611, figs. 1-3.

2005 Rosalina globularis d’Orbigny in Rasmussen, p. 94, pl. 14, fig. 1.

2012 Rosalina globularis d’Orbigny in Milker and Schmiedl, p. 98, fig. 22.15-16.

**Rosalina macropora (Hofker, 1951)** Figure 9 (HH, II)

1951 Discopulvinulina macropora in Hofker, p. 460, figs. 312, 313.

1960 Discopulvinulina macropora in Hofker, p. 253, pl. D, figs. 122 a-c.

1987 Rosalina globularis semiporata (Egger) in Wenger, p. 305, pl. 15, figs. 10-12.

1991 Rosalina macropora (Hofker) in Cimerman and Langer, p. 67, pl. 71, figs. 6-7.

**Rosalina orientalis (Cushman, 1915)**

1915 Discorbis globularis d’Orbigny (toothed variety) in Heron-Allen and Earland, p. 694, pl. LI, figs. 36-39.

1958 Rosalina orientalis (Cushman) in Collins, p. 404.

1987 Rosalina orientalis (Cushman) in Baccaert, p. 201, pl. 79, figs. 5-6.

1993 Rosalina orientalis (Cushman) in Hottinger et al., p. 111, pl. 143, figs. 7-9, pl. 144, figs. 1-2.

**Rosalina pellucida (Said, 1949)** Figure 9 (JJ, KK)

1949 Discorbis pellucidus in Said, p. 35, pl. 3, fig. 33 a-c.

1993 Rosalina pellucida (Said) in Hottinger et al., p. 111, pl. 144, figs. 3-6.

**Rosalina vilardeboana (d'Orbigny, 1839)** Figure 9 (LL, MM)

1839b Rosalina vilardeboana in d'Orbigny, p. 44, pl. 6, figs. 13-15.

1960 Rosalina vilardeboana (d'Orbigny) in Barker, pl. 86, fig. 9.

1991 Rosalina vilardeboana (d'Orbigny) in Cimerman and Langer, p. 67, pl. 72, figs. 1-2.

Superfamily GLABRATELLACEA Loeblich and Tappan, 1964

Family GLABRATELLIDAE Loeblich and Tappan, 1964

Genus Glabratella Dorreen, 1948

**Glabratella altispira (Buzas, Smith and Beem, 1977)** Figure 9 (Z, AA)

1977 Glabratella altispira in Buzas, Smith and Beem, p. 88, pl. 4, fig. 19-24

Genus CONORBELLA Hofker, 1951

**Conorbella imperatoria (d'Orbigny, 1846)**

1846 Rosalina imperatoria in d'Orbigny, p. 176, pl. 190, figs. 16-18.

1908 Discorbina imperatoria (d'Orbigny) in Sidebottom, p. 13, pl. 5, figs. 1, 2.

1985 Schackoinella imperatoria (d'Orbigny) in Papp and Schmid, p. 226, pl. 60, figs. 2-5.

1991 Conorbella imperatoria (d'Orbigny) in Cimerman and Langer, p. 68, pl. 73, figs. 4-7.

Genus GLABRATELLA Dorreen, 1948

**Glabratella erecta (Sidebottom, 1908)**

1908 Discorbina erecta in Sidebottom, p. 16, pl. 5, figs. 6, 7.

1991 Conorbella erecta (Sidebottom) in Cimerman and Langer, p. 68, pl. 72, figs. 6-8.

1993 Glabratella erecta (Sidebottom) in Sgarrella and Moncharmont-Zei, p. 220, pl. 18, figs. 7, 8.

2012 Glabratella erecta (Sidebottom) in Milker and Schmiedl, p. 102, fig. 23.12-13.

Superfamily SIPHONINACEA Cushman, 1927

Family CYMBALOPORIDAE Cushman, 1927

Subfamily CYMBALOPORINAE Cushman, 1927

Genus CYMBALOPORETTA Cushman, 1928

**Cymbaloporetta bradyi (Cushman, 1915)** Figure 10 (Q, R)

1915 Cymbalopora poeyi (d’Orbigny) var. bradyi in Cushman, p. 25, pl. 10, fig. 2; pl. 14, fig. 2.

1992b Cymbaloporetta bradyi (Cushman) in Hatta and Ujiié, p. 190, pl. 39, fig. 4; pl. 40, fig. 1.

1994 Cymbaloporetta bradyi (Cushman) in Loeblich and Tappan, p. 152, pl. 327, figs 8-10; pl. 328, figs

1-3.

1999 Cymbaloporetta bradyi (Cushman) in Hayward et al., p. 155; pl. 14, figs 28-29.

2012 Cymbaloporetta bradyi (Cushman) in Debenay, p. 236.

**Cymbaloporetta bulloides (d’Orbigny, 1839a)**

1839a Rosalina bulloides in d’Orbigny, p. 98, pl. 3, figs. 2-5.

1922b Tretomophalus bulloides (d’Orbigny) in Cushman, pp. 42-44, text-figs. 2, 3.

1987 Cymbaloporetta bulloides (d’Orbigny) in Loeblich and Tappan, pp. 170-171, pl. 649, figs. 11-15.

1991 Cymbaloporetta sp.1 in Cimerman and Langer, p. 72, pl. 80, figs. 1-5.

2012 Cymbaloporetta bulloides (d’Orbigny) in Milker and Schmiedl, p. 110, fig. 25.1-2.

Genus MILLETTIANA Banner, Pereira and Desai, 1985

**Millettiana millettii (Heron-Allen and Earland, 1915)**

1915 Cymbalopora millettii in Heron-Allen and Earland, p. 689, pI. 51, figs. 32-35.

1924 Tretomphalus millettii (Heron-Allen and Earland) in Cushman, p. 36, p1. II. fig. 4.

1985 Cymbaloporetta (Milleltiana) milleltii (Heron-Allen and Earland) in Banner et al., p. 170. pl. 4, figs. 110.

1987 Millelttalla milleltii (Heron-Allen and Earland) in Loeblich and Tappan, p. 591, pl. 648. figs. 7-II.

1994 Millelttalla milleltii (Heron-Allen and Earland) in Loeblich and Tappan, p. 153, pl. 329, figs. 1-2.

Family SIPHONINIDAE Cushman, 1927

Subfamily SIPHONININAE Cushman, 1927

Genus SIPHONINA Reuss, 1850

**Siphonina bradyana (Cushman, 1927)**

1927 Siphonina bradyana in Cushman, p. 11, pl. 1, fig. 4.

1978 Siphonina pulchra (Cushman) in Cheng and Zheng, (non S. pulchra Cushman, 1919b). p. 215, pl. 20, fig. 6.

1991 Siphonina bradyana in Van Marle, p. 223, pl. 19, figs. 13, 14.

1994 Siphonina bradyana (Cushman) in Loeblich and Tappan, p. 143, pl. 298, figs. 1-9.

**Siphonina reticulala (Czjzek, 1848)**

1848 Rotalina reticulata in Czjzek, p. 145, pl. 13, figs. 7-9.

1850 Siphonina fimbriata in Reuss, p. 372, pl. 47, fig. 6.

1931 Siphonina reticulata (Czjzek) in Cushman, p. 68, pl. 14, figs. 1 a-c.

1971 Siphonina reticulata (Czjzek) in Murray, p. 139, pl. 58, figs. 5-7.

1987 Siphonina reticulata (Czjzek) in Loeblich and Tappan, p. 571, pl. 624, figs. 4-6.

1991 Sipbonina reticulala (Czjzek) in Cimerman and Langer, p. 69, pl. 73, figs. 11-13.

Superfamily DISCORBINELLACEA Sigal in Piveteau, 1952

Family DISCORBINELLIDAE Sigal in Piveteau, 1952

Genus DISCORBINELLA Cushman and Martin, 1935

**Discorbinella bertheloti (d'Orbigny, 1839)** Figure 10 (E, F)

1839b Rosalina bertheloti in d'Orbigny, p. 135, pl. 1, figs 28-30.

1974 Discorbinella bertheloti (d'Orbigny) in Le Calvez, p. 59, pl. 14, figs. 1-4.

1991 Discorbinella bertheloti (d'Orbigny) in Cimerman and Langer, p. 70, pl. 86, figs. 1-4.

Family PSEUDOPARRELLIDAE Voloshinova, 1952

Subfamily PSEUDOPARRELLINAE Voloshinova, 1952

Genus FACETOCOCHLEA Loeblich and Tappan, n.gen.

**Facetocochlea pulchra (Cushman, 1933)** Figure 9 (BB, CC)

1933a Pulvinulinella pulchra in Cushman, p. 92, pI. 9. fig. 10.

1958 Pseudoparrella pulchra (Cushman) in Collins, p. 410.

1965 Epistominella pulchra (Cushman) in Todd, p. 31, pI. 10, figs. 3, 4.

1994 Facetocochlea pulchra (Cushman) in Loeblich and Tappan, p. 145, pl. 304, figs. 1-10.

Superfamily PLANORBULINACEA Schwager, 1877

Family PLANULINIDAE Bermúdez, 1952

Genus HYALINEA Hofker, 1951

**Hyalinea balthica (Schröter, 1783)**

1783 Nautilus balthicus in Schrater, p. 20, fig. 2 (fide Ellis and Messina, 1940).

1931 Anomalina balthica (Schröter) in Cushman, p. 108, pl. 19, fig. 3.

1971 Hyalinea balthica (Schröter) in Murray, p. 173, pl. 72, figs. 5-8.

1974 Hyalinea balthica (Schröter) in Colom, p. 151, fig. 37.

1987 Hyalinea balthica (Schröter) in Loeblich and Tappan, p. 580, pl. 632, figs. 5-8.

1991 Hyalinea balthica (Schröter) in Cimerman and Langer, p. 70, pl. 74, figs. 4-7, figs. 5-6.

Superfamily DISCORBINELLACEA Sigal, 1952

Family CIBICIDIDAE Cushman, 1927

Subfamily CIBICIDINAE Cushman, 1927

Genus CIBICIDES de Montfort, 1808

**Cibicides cf. mayori (Cushman, 1924)**

1924 cf. Truncatulina mayori in Cushman, p. 39, pl. 12, figs. 3, 4.

1965 cf. Cibicides mayori (Cushman) in Todd, p. 53, pl. 22, figs. 3, 4.

1975 cf. Cibicides(?) mayori (Cushman) in Perelis and Reiss, p. 77, pl. 3, figs. 1-7.

1993 cf. Cibicides? mayori (Cushman) in Hottinger et al., p. 116, pl. 152, figs. 1-6.

2012 Cibicides cf. mayori in Milker and Schmiedl, p. 106, fig. 24.10-11.

**Cibicides pseudolobatulus (Perelis and Reiss, 1975)**

1975 Cibicides pseudolobatulus in Perelis and Reiss, pp. 77-78, pl. 4, figs. 1-7, pl. 5, figs. 1, 2.

1993 Cibicides pseudolobatulus (Perelis and Reiss) in Hottinger et al., p. 116, pl. 152, figs. 7-11

.2012 Cibicides pseudolobatulus (Perelis and Reiss) in Milker and Schmiedl, fig. 21.12-13.

**Cibicides refulgens (de Montfort, 1808)**

1808 Cibicides refulgens in de Montfort, p. 123

1931 Cibicides refulgens (de Montfort) in Cushman, p. 116, pl. 21, fig.2

1987 Cibicides refulgens (de Montfort) in Loeblich and Tappan, p. 167, pl. 634, figs. 1-3

1991 Cibicides refulgens (de Montfort) in Cimerman and Langer, p. 70, pl. 75, figs. 5-9

2003 Cibicides refulgens (de Montfort) in Murray, p. 21, fig. 7, no. 1, 2

2005 Cibicides refulgens (de Montfort) in Rasmussen, p. 100, pl. 15, figs. 7, 8

2009 Cibicides refulgens (de Montfort) in Milker et al., p. 218, pl. 3, figs. 9, 10

2012 Cibicides refulgens (de Montfort) in Milker and Schmiedl, p. 105, figs. 24.14-16

Genus LOBATULA Fleming, 1828

**Lobatula lobatula (Walker and Jacob, 1798)**

1798 Nautilus lobatulus in Walker and Jacob, p. 642, pI. 14, fig. 36 (fide Ellis and Messina, 1940).

1896 Truncatulina lobatula (Walker and Jacob) in Deželić, p. 87.

1958 Cibicides lobatulus (Walker and Jacob) in Le Calvez, p. 188.

1960 Cibicides lobatulus (Walker and Jacob) in Barker, pI. 92, fig. 10, pl. 93, figs. 1,4,5, pl. 95, figs. 4, 5.

1970 Cibicides lobatulus (Walker and Jacob) in von Daniels, p. 89, pl. 8, fig. 3.

1974 Cibicides lobatulus (Walker and Jacob) in Colom, p. 147, figs. 29 a-g, i, fig. 30.

1984 Cibicides lobatulus (Walker and Jacob) in Reiss and Hottinger, fig. G. 29 d.

1979 Cibicides lobatulus (Walker and Jacob) in Alfirević, p. 148, pl. 33, fig. 3.

1987 Lobatula lobatula (Walker and Jacob) in Loeblich and Tappan, p. 583, pl. 637, figs. 10-13.

1991 Lobatula lobatula (Walker and Jacob) in Cimerman and Langer, p. 71, pl. 75, figs. 1-4.

Genus PARACIBICIDES Perelis and Reiss. 1975

**Paracibicides edomica (Perelis and Reiss, 1975)**

1975 Paracibicides edomica in Perelis and Reiss, p. 94, pl. 9, figs. 5,6, pl. 10, figs. 1-6, text fig. 8.

1987 Paracibicides edomica (Perelis and Reiss) in Loeblich and Tappan, p. 584. pl. 634, figs. 16-18.

1994 Paracibicides edomica (Perelis and Reiss) in Loeblich and Tappan, p. 150, pl. 322, figs. 1-3.

Family PLANORBULINIDAE Schwager, 1877

Subfamily PLANORBULININAE Schwager, 1877

Genus CIBICIDELLA Cushman, 1927

**Cibicidella variabilis (d’Orbigny, 1826)**

1826 Truncatulina variabilis in d’Orbigny, p. 279, no 8.

1974 Cibicidella variabilis (d’Orbigny) in Le Calvez, p. 96-98, pl. 26, fig. 4.

Genus PLANORBULINA d’Orbigny, 1826

**Planorbulina mediterranensis (d'Orbigny, 1826)** Figure 10 (M, N, O, P)

1826 Planorbulina mediterranensis in d'Orbigny, p. 280, no. 2.

1896 Planorbulina mediterranensis (d'Orbigny) in Deželić, p. 86.

1931 Planorbulina mediterranensis (d'Orbigny) in Cushman, p. 129, pl. 24, figs. 5-8.

1960 Planorbulina mediterranensis (d'Orbigny) in Barker, pI. 92, figs. 1-3.

1974 Planorbulina mediterranensis (d'Orbigny) in Colom, p. 158, figs. 39, 40.

1974 Planorbulina mediterranensis (d'Orbigny) in Le Calvez, p. 43, pI. 11, figs. 1-3.

1979 Planorbulina mediterranensis (d'Orbigny) in Alfirević, p. 150, pl. 33, fig. 4.

1987 Planorbulina mediterranensis (d'Orbigny) in Loeblich and Tappan, p. 588, pl. 645, figs. 1-4, pl. 646, figs. 1, 2.

1991 Planorbulina mcditerranensis (d'Orbigny) in Cimerman and Langer, p. 71, pl. 78, figs. 1-8.

Superfamily ASTERIGERINACEA d'Orbigny, 1839

Family ASTIGERINATIDAE Reiss, 1963

Genus ASTERIGERINATA Bermúdez, 1949

**Astigerinata mamilla (Williamson, 1858)** Figure 10 (S, T, U)

1858 Rotalina mamilla Williamson in Williamson, p. 54, pl. 4, figs. 109-111.

1991 Astigerinata mamilla (Williamson) in Cimerman and Langer, p. 73, pl. 82, figs. 1-4.

2008 Astigerinata mamilla (Williamson) in Abu-Zied et al., p. 53, pl. 3, figs. 8-9.

**Asterigerinata mariae (Sgarrella, 1990)**

1990 Asterigerinata mariae (Sgarrella) in type reference Amore et al., 1990, pp. 477-478, pl. 3, figs. 1-8.

1993 Asterigerinata mariae (Sgarrella) in Sgarrella and Moncharmont-Zei, p. 224, pl. 20, fig. 1.

2004 Asterigerinata mariae (Sgarrella) in Fiorini, p.50, pl. 1, figs. 16-18.

2009 Asterigerinata mariae (Sgarrella) in Milker et al., p. 218, pl. 3, figs. 21-23.

2012 Asterigerinata mariae (Sgarrella) in Milker and Schmiedl, p. 111, fig. 25.14-16.

Family AMPHISTEGINIDAE Cushman, 1927

Genus AMPHISTEGINA d’Orbigny, 1826

**Amphistegina lessonii (d’Orbigny, 1826)** Figure 11 (I, J)

1826 Amphistegina lessonii in d’Orbigny, p. 304.

1976 Amphistegina lessonii (d’Orbigny) in Larsen, p. 2, pl. 2, figs. 1-5, pl. 7, figs. 1, pl. 8, fig. 1.

1977 Amphistegina lessonii (d’Orbigny) in Larsen and Drooger, p. 225, fig. 1/2 a, b.

1984 Amphistegina lessonii (d’Orbigny) in Reiss and Hottinger, p. 217, Fig. G11, G12 d, e.

1993 Amphistegina lessonii (d’Orbigny) in Hottinger et al., p. 132, pl. 184, figs. 1-11, pl. 185, figs. 1-7.

**Amphistegina lobifera (Larsen, 1976)** Figure 11 (K, L)

1880 Amphistegina lessonii (d’Orbigny) in Möbius, p. 99, pl. 10, figs. 11-14, pl. 11, figs. 1-3.

1972 Amphistegina cf. radiata (Terquem) in Hansen and Reiss, pl. 10, fig. 3, 4.

1976 Amphistegina lobifera in Larsen, p. 4, pl. 3, figs. 1-5, pl. 7, fig. 3, pl. 8, fig. 3.

1977 Amphistegina lobifera in Larsen and Drooger, p. 225, fig. 1/1ab.

1984 Amphistegina lobifera (Larsen) in Reiss and Hottinger, p. 217, fig. G 11, G12a-c.

1988 Amphistegina lobifera (Larsen) in Morariu and Hottinger, p. 695, fig. 1A, B, fig. 2.

1993 Amphistegina lobifera (Larsen) in Hottinger et al., p. 133, pl. 186, figs. 1-11, pl. 187, figs. 1-6.

Superfamily NONIONACEA Schultze, 1854

Family NONIONIDAE Schultze, 1854

Subfamily NONIONINAE Schultze, 1854

Genus HAYNESINA Banner and Culver, 1978

**Haynesina depressula (Walker and Jacob, 1798)** Figure 10 (Y, Z)

1798 Nautilus depressulus in Walker and Jacob, p. 641, pI. 14, fig. 33 (fide Ellis and Messina, 1940).

1972 Nonion depressulum (Walker and Jacob) in Rosset-Moulinier, p. 186, pI. 21, figs. 1-4, pI. 22, figs. 1, 2 .

1976 Nonion depressulum (Walker and Jacob) in Hansen and Lykke-Andersen, p. 21, pI. 19, figs. 3. 6.

1978 Haynesina depressula (Walker and Jacob) in Banner and Culver, p. 200, pI. 10, figs. 1-8.

1991 Haynesina depressula (Walker and Jacob) in Cimerman and Langer, p. 81, pl. 83, figs. 1-4.

**Haynesina simplex (Cushman, 1933b)** Figure 10 (AA, BB)

1933b Elphidium simplex in Cushman, p. 52, pl. 12, figs. 8, 9.

1991 Haynesina sp. 2 in Cimerman and Langer, p. 82, pl. 83, figs. 9, 10.

2005 Haynesina depressulum simplex (Cushman) in Debenay et al., p. 336, pl. 3, fig. 16.

2010 Haynesina sp. 2 in Milker, p. 125, pl. 6, fig. 8.

2012 Haynesina simplex (Cushman) in Milker and Schmiedl, p. 112, fig. 25.19-20.

Genus NONION de Montfort, 1808

**Nonion fabum (Fichtel and Moll, 1798)** Figure 10 (EE, FF)

1798 Nautilus faba in Fichtel and Moll, p. 103, pl. 19 b, c.

1884 Nonionina boueana d'Orbigny in Brady, p. 729, pl. 109, figs. 12, 13.

1987 Nonion fabum (Fichtel and Moll) in Loeblich and Tappan, p. 179, pl. 690, figs. 1-7.

1994 Nonion fabum (Fichtel and Moll) in Jones, p. 108, pl. 109, figs. 12, 13 [cop. Brady, 1884, figs. 12, 13].

2004 Nonion fabum (Fichtel and Moll) in Mendes et al., p. 178. pl. 1, fig. 10.

2005 Nonion fabum (Fichtel and Moll) in Rasmussen, p. 102, pl. 16, fig. 5.

2012 Nonion fabum (Fichtel and Moll) in Milker and Schmiedl, p. 112, fig. 25.22-24.

Genus NONIONELLA Cushman, 1926

**Nonionoides grateloupi (d'Orbigny, 1826)** Figure 10 (II, JJ)

1826 Nonionina grateloupi in d’Orbigny, p. 294.

1839 Nonionina grateloupi in d’Orbigny, p. 46, pI. 6, figs. 6. 7.

1939 Nonionina grateloupi (d'Orbigny) in Cushman, p. 21, pl. 6. figs. 1-7.

1975 Nonionoides grateloupi (d'Orbigny) in Saidova, p. 248, pI. 67, fig. 5.

1987 Nonionoides grateloupi (d'Orbigny) in Loeblich and Tappan, p. 618, pI. 692. figs. 7-14.

1994 Nonionoides grateloupi (d'Orbigny) in Loeblich and Tappan, p. 158, pl. 342, figs. 1-5.

**Nonionella turgida (Williamson, 1858)** Figure 10 (GG, HH)

1858 Rotalina turgida in Williamson, p. 50, pl. 4, figs. 95-97.

1960 Nonionella turgida (Williamson) in Barker, pl. 109, figs. 17-19.

1971 Nonionella turgida (Williamson) in Murray, p. 193, pl. 81, figs. 1-5.

1991 Nonionella turgida (Williamson) in Cimerman and Langer, p. 74, pl. 84, figs. 6-8.

Subfamily ASTRONONIONINAE Saidova, 1981

Genus ASTRONONION Cushman and Edwards, 1937a

**Astrononion stelligerum (d'Orbigny, 1839)** Figure 10 (V, W, X)

1839b Nonionina stelligera in d'Orbigny, p. 128, pl. 3, fig. 12.

1930 Nonion stelligerum (d'Orbigny) in Cushman, p. 7, pI. 8, figs. 8-12, pl. 3, figs. 1-3.

1937a Astrononion stelligerum (d'Orbigny) in Cushman and Edwards, p. 31, pl. 3, fig. 7 a.

1960 Astrononion stelligerum (d'Orbigny) in Barker, pI. 109, figs. 3, 4.

1974 Astrononion stelligerum (d'Orbigny) in Le Calvez, p. 37, pl. 9, figs. 1-4.

1991 Astrononion stelligerum (d'Orbigny) in Cimerman and Langer, p. 74, pl. 84, figs. 13-15.

Subfamily PULLENIINAE Schwager, 1877

Genus MELONIS de Montfort, 1808

**Melonis affinis (Reuss, 1851)** Figure 10 (CC, DD)

1851 Nonionina affinis in Reuss, p. 72, pl. 5, fig. 32.

1884 Nonionina umbilicatula (Montagu) in Brady, p. 726, pl. 109, figs. 8, 9.

1978 Nonion affine (Reuss) in Boltovskoy, p. 162, pl. 5, figs. 1, 2.

1990 Melonis affinis (Reuss) in Sprovieri and Hasegawa, p. 457, pl. 3, figs. 11, 12.

1994 Melonis affinis (Reuss) in Jones, p. 107, pl. 109, figs. 8, 9 [cop. Brady, 1884, figs. 8, 9].

2008 Melonis affinis (Reuss) in Abu-Zied et al., p. 53, pl. 3, figs. 14, 15.

2009 Melonis pompilioides (Fichtel and Moll) in Avsar et al., p. 135, pl. 3, figs. 10, 11.

2009 Melonis affinis (Reuss) in Milker et al., p. 218, pl. 3, fig. 20.

2012 Melonis affinis (Reuss) in Milker and Schmiedl, p. 115, fig. 26.9-10.

Superfamily NUMMULITACEA de Blainville, 1827

Family NUMMULITIDAE de Blainville, 1827

Genus ASSILINA d'Orbigny 1839a

**Assilina ammonoides (Gronovius, 1781)**

1781 Nautilus ammonoides in Gronovius, p. 282. pI. 19. figs. 5, 6.

1884 Operculina ammonoides (Gronovius) in Brady, p. 745, pl. 112, figs. 1, 2.

1941 Operculina ammonoides (Gronovius) in Leroy, p. 78, pl. 6, figs. 24, 25.

1961 Neooperculinoides ammonoides (Gronoviu) in Golev, p. 114.

1987 Assifina ammonoides (Gronovius) in Loeblich and Tappan, p. 682, pl. 804, figs. 1-7.

1994 Assilina ammonoides (Gronovius) in Loeblich and Tappan, p. 170, pl. 387, figs. 7-9. Pl. 388, figs. 1-4.

Family TRICHOHYALIDAE Saidova, 1981

Genus BUCELLA Anderson, 1952

**Buccella frigida (Cushman, 1922)**

1922a Pulvinulina frigida in Cushman, p. 12.

1865 Pulvinulina frigida (Cushman) in Parker and Jones, 1865, pl. 14, Figs. 14, 15a–b, 17.

1931 Eponides frigidus in Cushman, 1931, p. 45

1952 Buccella frigida in Andersen, p. 144, Figs. 4-6

Family ROTALIIDAE Ehrenberg, 1839

Subfamily AMMONIINAE Saidova, 1981

Genus AMMONIA Brünnich, 1772

**Ammonia beccarii (Linnaeus, 1758)** Figure 11 (A, B)

1758 Nautilus beccarii in Linnaeus, p. 710 (fide Ellis and Messina, 1940)

1858 Rotalina beccarii (Linnaeus) in Williamson, p. 48, pl. 4, figs. 90-92.

1931 Rotalia beccarii (Linnaeus) in Cushman, p. 58, pl. 12, figs. 1-7, pl. 13, figs. 1, 2.

1979 Ammonia beccarii (Linnaeus) in Alfirevic, p. 126, pl. 27, fig. 1.

1980 Ammonia beccarii (Linnaeus) in Billman and aI., p. 85, p. 1, figs. 1-10, pl. 2, figs. 1-6.

1987 Ammonia beccarii (Linnaeus) in Loeblich and Tappan, p. 664, pl. 767, figs. 1-7.

1991 Ammonia beccarii (Linnaeus) in Cimerman and Langer, p. 76, pl. 87, figs. 3-4.

**Ammonia caspica (Shchedrina, 1975)**

1968 Ammonia beccarii caspica in Mayer, p. 28, fig. 48.

1975 Ammonia neobeccarrii caspica in Shchedrina and Mayer, p. 255, figs. 1-8.

1989 Ammonia neobeccarrii caspica in Yanko, p. 177-179, figs. 1-12.

**Ammonia convexa (Collins, 1958)** Figure 11 (C, D)

1958 Streblus convexus in Collins, p. 414, pl. 5, fig. 10.

1994 Ammonia convexa (Collins) in Loeblich and Tappan, p. 165, pl. 369, figs. 1-10

**Ammonia parkinsoniana (d'Orbigny, 1839)** Figure 11 (E, F)

1839a Rosalina parkinsoniana in d'Orbigny, p. 99, pl. 4, figs. 25-27.

1977b Ammonia parkinsoniana (d'Orbigny) in Le Calvez, p. 92, pl. 11, figs. 1-3.

1991 Ammonia parkinsoniana (d'Orbigny) in Cimerman and Langer, p. 76, pl. 87, figs. 7-9.

**Ammonia tepida (Cushman, 1926)** Figure 11 (G, H)

1926 Rotalia beccarii (Linnaeus) var. tepida in Cushman, p. 79, pl. 1 (fide Ellis and Messina, 1940)

1931 Rotalia beccarii (Linnaeus) var. tepida Cushman in Cushman, p. 61, pl. 13, figs. 3 a-c

1965 Streblus beccarii tepida (Cushman) in Todd, p. 29, pl. 6, fig. 1, pl. 7, fig. 2

1972 Ammonia beccarii (Linnaeus) var. tepida (Cushman) in Rosset-Moulinier, p. 174

1991 Ammonia tepida (Cushman) in Cimerman and Langer, p. 76, pl. 87, figs. 10-12

Family ELPHIDIIDAE Galloway, 1933

Subfamily ELPHIDIINAE Galloway, 1933

Genus ELPHIDIUM de Montfort, 1808

**Elphidium advenum (Cushman, 1922)**

1922b Polystomella advena in Cushman, p. 56, pl. 9, figs. 11, 12.

1933b Elphidium advenum (Cushman) in Cushman, p. 50, pl. 12, figs. 1-3.

1958 Elphidium advena (Cushman) in Parker, p. 269, pl. 4, figs. 3, 4.

1993 Elphidium advenum (Cushman) in Hottinger et al., p. 146, pl. 207, figs. 1-7.

1993 Elphidium punctatum (Terquem) in Sgarrella and Moncharmont Zei, p. 230, pl. 21, figs. 3, 4.

2005 Elphidium advenum (Cushman) in Rasmussen, p. 108, pl. 18, figs. 13-15.

2012 Elphidium advenum (Cushman) in Milker and Schmiedl, p. 119, fig. 27.7-8.

**Elphidium complanatum (d’Orbigny, 1839b)**

1839b Polystomella complanata in d’Orbigny, p. 129, pl. 2, figs. 35, 36.

1958 Elphidium complanatum (d’Orbigny) in Parker, p. 270, pl. 4, fig. 5.

1933b Elphidium jenseni (Cushman) in Cushman, p. 48, pl. 11, figs. 6, 7.

1991 Elphidium jenseni (Cushman) in Cimerman and Langer, p. 78, pl. 92, figs. 1-3.

1993 Elphidium jenseni (Cushman) in Hottinger et al., p. 148, pl. 211, figs. 8-14.

1993 Elphidium complanatum (d’Orbigny) in Sgarrella and Moncharmont-Zei, p. 228, pl. 20, figs. 9, 10.

2005 Elphidium jenseni (Cushman) in Debenay et al., p. 336 pl. 3, figs. 26, 27.

2005 Elphidium complanatum (d’Orbigny) in Rasmussen, p. 109, pl. 19, fig. 2.

2006 Elphidium complanatum (d’Orbigny) in Avsar et al., p. 134, pl. 3, fig. 19.

2012 Elphidium complanatum (d’Orbigny) in Milker in Schmiedl, p. 120, fig. 27.9-10.

**Elphidium crispum (Linnaeus, 1758)** Figure 11 (M, N)

1758 Nautilus crispus in Linnaeus, p. 709 (fide Ellis and Messina, 1940).

1960 Elphidium crispum (Linnaeus) in Barker, pl. 110, figs. 6, 7.

1970 Elphidium crispum (Linnaeus) in von Daniels, p. 87, pI. 7, fig. 7.

1974 Elphidium crispum (Linnaeus) in Colom, p. 143, figs. 26 e-j, m, n.

1976 Elphidium crispum (Linnaeus) in Hansen and Lykke-Andersen, p. 6, pI. 1, figs. 10-12, p. 2, figs. 1, 2.

1991 Elphidium crispum (Linnaeus) in Cimerman and Langer, p. 77, pl. 90, figs. 1-6.

**Elphidium depressulum (Cushman, 1933a)** Figure 11 (O, P)

1933a Elphidium advenum (Cushman) var. depressulum in Cushman, p. 51, pI. 12, figs. 4 a, b.

1991 Elphidium depressulum (Cushman) in Cimerman and Langer, p. 78, pl. 90, figs. 7-8.

**Elphidium gerthi (van Voorthuysen, 1957)**

1957 Elphidium gerthi in van Voorthuysen, p. 32 pI. 23, fig. 12 (fide Ellis and Messina, 1940).

1971 Elphidium gerthi (van Voorthuysen) in Murray, p. 161, pI. 67, figs. 1-7.

1972 Cribrononion gerthi (van Voorthuysen) in Rosset-Moulinier, p. 188, pl. 25, figs. 1,2,4.

1976 Elphidium gerthi (van Voorthuysen) in Hansen and Lykke-Andersen, p. 10, pl. 5, figs. 7-12.

1991 Elphidium gerthi (van Voorthuysen) in Cimerman and Langer, p. 78, pl. 91, figs. 1-2.

**Elphidium granosum (d’Orbigny, 1846)**

1846 Nonionina granosa in d’Orbigny, p. 110, pl. 5, fig. 19-20.

1958 Elphidium granosum (d’Orbigny) in Parker, p. 270, pl. 4, figs. 10, 11.

1987 Elphidium granosum forma granosum (d’Orbigny) in Jorissen, p. 39, pl. 2, figs. 1, 2.

1993 Elphidium granosum (d’Orbigny) in Sgarrella and Moncharmont Zei, p. 229, pl. 21, figs. 1, 2.

1995 Cibroelphidium granosum (d’Orbigny) in Coppa and Di Tuoro, p. 172, pl. 4, fig. 2.

2004 Porosononion granosum (d’Orbigny) in Chendes et al., p. 76, pl. 4, fig. 7.

2005 Elphidium granosum (d’Orbigny) in Rasmussen, p. 110, pl. 19, fig. 8.

2009 Porosononion subgranosum (Egger) in Avsar et al., p. 135, pl. 3, fig. 17, 18.

2009 Elphidium granosum (d’Orbigny) in Frezza and Carboni, p. 57, pl. 2, fig. 5.

2010 Elphidium granosum (d’Orbigny) in Milker, p. 129, pl. 7, fig. 14.

**Elphidium incertum (Williamson, 1858)** Figure 11 (Q, R)

1858 Polystomella umbilicula Walker var. incertum in Williamson, p. 44, pl. 3, fig. 82.

1884 Polystomella striatopunctata Fichtel and Moll in Brady, p. 733, pl. 109, fig. 23.

1930 Elphidium incertum (Williamson) in Cushman, p. 18, pl. 7, figs. 4-9 [fig. 4: cop. Williamson, 1858, fig. 82; fig. 5: cop. Brady, 1884, fig. 23].

1993 Elphidium incertum (Williamson) in Sgarrella and Moncharmont Zei, p. 229, pl. 21, fig. 5.

1994 Cribrononion incertum (Williamson) in Jones, p. 108, pl. 109, fig. 23 [cop. Brady, 1884, fig. 23].

2005 Elphidium excavatum (Terquem) in Rasmussen, p. 110, pl. 19, figs. 6, 7.

2012 Elphidium incertum (Williamson) in Milker and Schmiedl, p. 121, fig. 27.19-20.

**Elphidium macellum (Fichtel and Moll, 1798)**

1798 Nautilus macellus in Fichtel and Moll, p. 66, pl. 10, figs. e-g.

1884 Polystomella macella (Fichtel and Moll) in Brady, p. 737, pl. 110, figs. 8, 11.

1914 Elphidium macellum (Fichtel and Moll) in Cushman, p. 33, pl. 18, fig. 3.

1987 Elphidium crispum forma macellum (Fichtel and Moll) in Jorissen, p. 41, pl. 3, fig. 9.

1987 Elphidium macellum (Fichtel and Moll) in Loeblich and Tappan, p. 199, pl. 789, figs. 1-5.

1993 Elphidium macellum (Fichtel and Moll) in Sgarrella and Moncharmont-Zei, p. 229, pl. 20, fig. 12.

1994 Elphidium macellum (Fichtel and Moll) in Jones, p. 109, pl. 110, figs. 8, 11 [cop. Brady, 1884, figs. 8, 11].

2002 Elphidium macellum (Fichtel and Moll) in Kaminski et al., p. 179, pl. 5, fig. 11.

2004 Elphidium macellum (Fichtel and Moll) in Fiorini, p. 54, pl. 3, figs. 1, 2.

2005 Elphidium macellum (Fichtel and Moll) in Rasmussen, p. 110, pl. 19, fig. 10.

2012 Elphidium macellum (Fichtel and Moll) in Milker and Schmiedl, p. 122, fig. 27.21-22.

**Elphidium margaritaceum (Cushman, 1930)**

1930 Elphidium advenum (Cushman) var. margaritaceum in Cushman, p. 25, pl. 10, fig. 3.

1957 Elphidium margaritaceum (Cushman) in Voorthuysen, p. 32, pl. 23, fig. 13.

1976 Elphidium margaritaceum (Cushman) in Hansen and Lykkc-Andersen, p. 8, pl. 3, figs. 2-6.

1991 Elphidium margaritaceum (Cushman) in Cimerman and Langer, p. 79, pl. 92, figs. 4-6.

Family ROTALIIDAE Ehrenberg, 1839

Subfamily CUVILLIERININAE Loeblich and Tappan, 1964

Genus PARAROTALIA Le Calvez, 1949

**Pararotalia cf. P. socorroensis (McCulloch 1977)**

1977 cf. Praeglobotruncana? socorroensis in McCulloch, p. 424, pl. 178, fig. 4, pl. 179, figs. 1,3.

1993 Pararotalia cf. P. socorroensis (McCulloch) in Hottinger et al., p. 141, pl. 200, figs. 1-11.

Order CARTERINIDA Loeblich and Tappan, 1981

Suborder, CARTERININA Loeblich and Tappan, 1981

Family CARTERINIDAE Loeblich and Tappan, 1955

Genus CARTERINA Brady, 1884

**Carterina spiculotesta (Carter, 1877)**  Figure 8 (Z, AA)

1877 Rotalia spiculotesta in Carter, p. 470; pl. 16.

1884 Carterina spiculotesta (Carter) in Brady, p. 346; pl. 41, figs 7-10.

2012 Carterina spiculotesta (Carter) in Debenay, p.77.

REFERENCES

Abu-Zied RH, Rohling EJ, Jorissen FJ, Fontanier C, Casford JSL., Cooke S. 2008. Benthic foraminiferal response to changes in bottom-water oxygenation and organic carbon flux in the eastern Mediterranean during LGM to Recent times. Marine Micropaleontology 67: 46-68.

Akimoto K. 1990. Distribution of Recent benthic foraminiferal faunas in the Pacific off Southwest Japan and around Hachijojima Island. Science Report of the Tohoku University, Series 2 60: 139-223

Alberola C, Usera J, Garcia-Forner A. 1991. Distribucion de las Tanatocenosis de foraminiferos arenaceos en el Puerto de Los Alfaques (Terragona). Revista Espanola de Paleontologia Extraordinario: 77-85.

Alfirevic S. 1979. Rasprostranjenost i ekologija foraminifera otvorenog Jadrana. PhD Thesis, University of Zagreb, Zagreb, Croatia.

Amore FO, Barra D, Ciampo G, Ruggiero Taddei E, Russo GF, Sgarrella F. 1990. Paleoecologia dei depositi terrazzati della Starza (Pozzuoli). In: Robba E, ed., Proceedings of the Fourth Symposium of Ecology and Paleoecology of Benthic Communities. Museo Regionale di Scienze Naturali, Torino, pp. 455-499.

Andersen HV. 1952. Buccella, a new genus of the rotalid foraminifera. Journal of the Washington Academy of Sciences 42: 143-151.

Avsar N, Aksu A, Dincer F. 2006. Benthic foraminiferal assemblage of Erdek Bay (SW Marmara Sea).Yerbilimleri 27: 125-141.

Avsar N, Meric E, Cevik MG, Dincer F. 2009. Recent benthic foraminiferal assemblages on the continental shelf off the Büyük Menderes river delta (W Turkey).Yerbilimleri 30: 127-144.

Baccaert J. 1987. Distribution patterns and taxonomy of Foraminifera in the Lizard Island Reef Complex, northern Barrier Reef, Australia. PhD Thesis, Liege, C.A.P.S.

Bagg RM. 1898. The Tertiary and Pleistocene Foraminifera of the middle Atlantic slope. Bulletin of American Paleontology 2: 3-54.

Banner FT, Culver SJ. 1978. Quaternary Haynesina n. gen. and Paleogene Protelphidium Haynes; their morphology, affinities and distribution. Journal of Foraminiferal Research 8: 177-207.

Banner FT, Pereira CPG, Desai D. 1985. "Tretomphaloid" float chambers in the Discorbidae and Cymbaloporidae. Journal of Foraminferal Research 15: 159-174.

Barker RW. 1960. Taxonomic notes on the species figured by H.B. Brady in his report on the foraminifera dredged by H.M.S. Challenger during years 1873-1876. Society of Economic Paleontologists and Mineralists, Special Publication Number 9: 1-235.

Barras C, Jorissen FJ, Labrune C, Andral B, Boissery P. 2014. Live benthic foraminiferal faunas from the French Mediterranean Coast: Towards a new biotic index of environmental quality. Ecological indicators 36: 719-743.

Belford DJ. 1966. Miocene and Pliocene smaller foraminifera from Papua and New Guinea. Australian Bureau of Mineral Resources, Geology and Geophysics. Australia Bulletin 79: 1-306.

Bender H. 1989. Gehäuseaufbau, Gehäusegenesc and Biologic agglutinierter Foraminiferen (Sarcodina. Textulariina. Jahrbuch der Geologischen Bundesanstalt 132: 259-347.

Bermudez PJ. 1949. Pavoninoides, a new genus of the Miliolidae from Panama. Contributions of the Cushman Laboratory for Foraminiferal Research 25: 58.

Bermudez PJ. 1952. Estudio sistematico de los foraminiferos rotaliformes. Boletin de Geologia 2(4):1-230.

Berthelin G. 1880. Memoire sur les Foraminiféres fossils de l´Etage Albien de Moncley (Doubs). Mémoires de la Société Géologique de France, Series 3 1(5): 1-84.

Billman H, Hottinger L, Oesterle H. 1980. Neogene to Recent rotaliid foraminifera from the Indopacific Ocean; their canal system, their classification and their stratigraphic use. Abhandlungen der Schwizerischen Palaontologischen Gesellschaft 101: 71-113.

Boltovskoy E. 1978. Estudio bioestratigrafico y paleontologico (Foraminiferos bentonicos) del Cenozoico Superior al este de las Islas Malvinas (DSDP, Crucero 36, Sitios 327 y 329). Revista Museo Argentino de Ciencias Naturales "Bernardino Rivadavia" e Imtituto Nacional de Investigacion de las Ciencias Naturales, Geologia 8(2): 19-70.

Brady HB. 1879. Notes on some reticularian Rhizopoda of the "Challenger" expedition, Part 1. Quaternary Journal of the Microscopical Science 19 (new series): 20-63.

Brady HB. 1881. Notes on some reticularian Rhizopoda of the "Challenger" expedition, Part 3. Quaternary Journal of the Microscopical Science 21(new series): 31-71.

Brady HB. 1884. Report on the foraminifera dredged by H.M.S. Challenger during the years 1873-1876. Report on the scientific results of the voyage of H.M.S. Challenger during the years 1873-1876, Zoology 9: 1-814.

Broennimann P. 1976. Two new genera of Recent Trochamminidae (Foraminiferida). Archives des Sciences, Genève 29: 215-218.

Broennimann P. 1979. Recent benthonic foraminifera from Brasil. Morphology and ecology. Part IV: Trochamminids from the Campos Shelf with description of Paratrochammina, Paliiontologische Zeitschrift 53: 5-25.

Broennimann P, Beurlen G. 1977. Recent benthonic foraminifera from Brazil. Morphology and ecology, Part I. Archives des Sciences, Genève 30: 77- 89.

Broennimann P, Whittaker JE. 1983. A lectotype for Deuterammina (Deuterammina) rotaliformis (Heron-Allen and Earland) and new trochamminids from E. Ireland (Protozoa: Foraminiferida). Bulletin of the British Museum (National History), Zoology Series 45: 347-358.

Brünnich MT. 1771. Bruennichii Zoologiae fundamenta. Hafniae et Lipsiae: Grunde i Dyeloeren.

Bruguiere JG. 1792. Encyclopedie methodique. Histoire naturelle des Verso Tome Premier. Paris, A-Cone, Panckoucke.

Buzas MA, Smith RK, Beem KA. 1977. Ecology and systematics of foraminifera in two Thalassia habitats, Jamaica, West Indies. Smithsonian Contributions to Paleobiology 31: 1-139. https://doi.org/10.5479/si.00810266.31.1

Carter HJ. 1877. On a Melobesian form of foraminifera (Gypsina melobesioides. mihi): and further observations on Carpenteria monticularis. Annals and Magazine of Natural History 20: 172-176.

Chapman F. 1907. Recent Foraminifera of Victoria: Some littoral gatherings. Journal Quekett Microscopical Club 10(2): 117-146.

Chapman E, Parr WJ, Collins AC. 1934. Tertiary foraminifera of Victoria, Australia-The Balcombian deposits of Port Phillip, Part III, Journal of the Linnaean Society of London, Zoology 38: 553-577.

Chendes C, Kaminiski MA, Filipescu S, Aksu AE, Yasar D. 2004. The response of modern benthic foraminiferal assemblages to water-mass properties along the southern shelf of Marmara Sea. Acta Palaeontologica Romaniae 4: 69-80.

Cheng TC, Zheng SY. 1978. The Recent foraminifera of the Xisha Islands, Guangdong Province, China I. Studia Marine Sinica 12: 149-266.

Cherif OH. 1970. Die Miliolacea der Westktiste von Naxos (Griechenland) und ihre Lebensbereiche. Unpublished PhD-Thesis, Fakultät der Natur- und Geisteswissenschaften, Technische Universität Clausthal, Germany.

Cimerman F, Langer MR. 1991. Mediterranean Foraminifera Slovenska Akademija Znanosti in Umetnosti. Academia Scientiarum et Artium Slovenica, Ljubljana.

Collins AC. 1958. Foraminifera, in Great Barrier Reef Expedition 1928-29, Scientific Reports, British Museum (Natural History) 6(6): 335-437.

Colom G. 1974. Foraminiferos ibericos. Introduccion al estudio delas especies bentonicas recientes. Investigacion Pesquera, Consejo Superior de Investigaciones Cientıficas, Patronato Juan de la Cierva, Barcelona.

Coppa MG, Di Tuoro A. 1995. Preliminary data on the Holocene foraminifera of the Cilento continental shelf (Tyrrhenian Sea). Revista Espanola de Paleontologia 10: 161-174.

Costa OG. 1856. Paleontologia del regno di Napoli, Parte II. Atti dell´Accademia Pontaniana Napoli 7(2): 113-378.

Cushman JA. 1910. A monograph of the foraminifera of the North Pacific Ocean, Part 1: Astrorhizidae and Lituolidae. Bulletin of the United States National Museum 71(1): 1-134.

Cushman JA. 1911. A monograph of the foraminifera of the North Pacific Ocean, Part 2: Textularidae. Bulletin of the United States National Museum 71(2): 1-108.

Cushman JA. 1913. A monograph of the foraminifera of the North Pacific Ocean, Part 3, Lagenidae. Bulletin of the United States National Museum 71(3): 1-122.

Cushman JA. 1914. A monograph of the foraminifera on the North Pacific Ocean, Part 4: Chilostomellidae, Globigerinidae, Nummulitidae. Bulletin of the United States National Museum 71(4): 1-46.

Cushman JA. 1915. A monograph of the foraminifera of the North Pacific Ocean, Part 5: Rotaliidae. Bulletin of the United States National Museum 71(5): 1-87.

Cushman JA. 1917. A monograph of the foraminifera of the North Pacific Ocean, Part 6: Miliolidae. Bulletin of the United States National Museum 71(6): 1-108.

Cushman JA. 1918. The foraminifera of the Atlantic Ocean, Part 1: Astrorhizidae. Bulletin of the United States National Museum 104(1): 1-111.

Cushman JA. 1920. The foraminifera of the Atlantic Ocean, Part 2: Lituolidae. Bulletin of the United States National Museum 104(2): 1-111.

Cushman JA. 1921. Foraminifera of the Philippine and adjacent seas. Bulletin of the United States National Museum 4(100): 1–608.

Cushman JA. 1922a. The foraminifera of the Atlantic Ocean, Part 3: Textularidae. Bulletin of the United States National Museum 104(3): 1-143.

Cushman JA. 1922b. The foraminifera of the Byram Calcareous Marl at Byram, Mississipi, p. 82-281. In White, D. (ed.), Shorter Contributions to General Geology. US Geological Survey Professional Paper 129E, Washington.

Cushman JA. 1923. The foraminifera of the Atlantic Ocean, Part 4: Lagenidae. Bulletin of the United States National Museum 104(4): 1-129.

Cushman JA. 1924. Samoan Foraminifera. Carnegie Institution of Washington, no. 342, Department of Marine Biology 21: 1-75.

Cushman JA. 1926. Foraminifera of the typical Monterey of California. Contributions from the Cushman Laboratory for Foraminiferal Research 2: 53-69.

Cushman JA. 1927. An outline of a re-classification of the foraminifera. Contributions from the Cushman Laboratory for Foraminiferal Research 3: 1-105.

Cushman JA. 1928. Additional genera of the foraminifera. Contributions from the Cushman Laboratory for Foraminiferal Research 4: 1-8.

Cushman JA. 1929. The foraminifera of the Atlantic Ocean, Part 6: Miliolidae, Ophthalmidiidae, Fischerinidae. Bulletin of the United States National Museum 104(6): 1-129.

Cushman JA. 1930a. A resume of new genera of the foraminifera erected since early 1928. Contributions from the Cushman Laboratory for Foramimferal Research 6: 73-94.

Cushman JA. 1930b. The foraminifera of the Atlantic Ocean, Part 7. Nonionidae, Camerinidae, Peneroplidae and Alveolinellidae. Bulletin United States National Museum 104(7): 1-79.

Cushman JA. 1931. The foraminifera of the Atlantic Ocean, Part 8: Rotaliidae, Amphisteginidae, Calcarinidae, Cymbalporettidae, Globorotallidae, Anomalinidae, Planorbulinidae, Pupertiidae and Homotremidae. Bulletin of the United States National Museum 104(8): 1-179.

Cushman JA. 1932. The foraminifera of the tropical Pacific collections of the "Albatross," 1899-1900, Part 1: Astrorhizidae to Trochamminidae. Bulletin of the United States National Museum 161: 1-88.

Cushman JA. 1933a. Foraminifera their classification and economic use. Special Publications Cushman Laboratory for Foraminiferal Research 4: 1-349.

Cushman JA. 1933b. Some new foraminiferal genera. Contributions from the Cushman Laboratory for Foraminiferal Research 9: 32-38.

Cushman JA. 1934a. Notes on the genus Tretomphalus, with descriptions of some new species and a new genus, Pyropilus, Contributions from the Cushman Laboratory for Foraminiferal Research 10: 79-101.

Cushman JA. 1934b. Notes on the genus Tretomphalus, with descriptions of some new species and a new genus, Pyropilus. Contributions from the Cushman Laboratory for Foraminiferal Research 10(4): 79-101.

Cushman JA. 1936. New genera and species of the families Verneuilinidae and Valvulinidae and of the subfamily Virgulininae. Cushman Laboratory for Foraminiferal Research, Special Publication 6: 1-71.

Cushman JA. 1937a. A monograph of the foraminiferal family Valvulinidae. Special Publications Cushman Laboratory for Foraminiferal Research 8: 1-210.

Cushman JA. 1937b. A monograph of the subfamily Virgulininae of the foraminiferal family Buliminidae: Special Publications, Cushman Laboratory for Foraminiferal Research 9(15): 1–228.

Cushman JA. 1939. A monograph of the foraminiferal family Nonionidae. Professional Papers US. Geological Survey 191: 1-100.

Cushman JA. 1942. The foraminifera of the tropical Pacific collections of the "Albatross," 1899-1900. Part 3. Heterohelicidae and Buliminidae. Bulletin United States National Museum 161: 1-67.

Cushman JA. 1944. Foraminifera from the shallow water of the New England coast, Special Publications Cushman Laboratory for Foraminiferal Research 12: 1-37.

Cushman JA. 1945. Foraminifera of the United States Antarctic Service Expedition 1939-1941. Reports on Scientific Results of the Antarctic Service Expedition, 1939-1941. Proceedings of the American Philosophical Society 89: 285-288.

Cushman JA, Edwards PG. 1937. Astrononion a new genus of the foraminifera, and its species. Contributions from the Cushman Laboratory for Foraminiferal Research 13: 29-36.

Cushman JA, Martin LT. 1935. A new genus of foraminifera, Discorbinella, from Monterey Bay, California. Contributions from the Cushman Laboratory for Foraminiferal Research 11: 89-90.

Cushman JA, Parker FL. 1931. Recent foraminifera from the Atlantic coast of South America. Proceedings U.S. National Museum 80(3): 1-24.

Cushman JA, Parker FL. 1947. Bulimina and related foraminiferal genera, Professional Papers U S. Geological Survey 210-D: 55-176.

Cushman JA, Wickenden RTD. 1928. A new foraminiferal genus from the Upper Cretaceous, Contributions from the Cushman Laboratory for Foraminiferal Research 4: 12-13.

Czjzek J. 1848. Beitrag zur Kenntnis der fossilen Foraminiferen des Wiener Beckens. Haidingers Naturwissenschaftliche Abhandlungen Wien 2: 137-150.

Daniels CV. 1970. Quantitative ökologische Analyse der zeitlichen und räumlichen Verteilung rezenterForaminiferen im Limskikanal bei Rovinj (nördlicheAdria). Göttinger Arbeiten zur Geologie und Paläontologie 8:1-109.

Debenay JP, Millet B, Angelidis MO. 2005. Relationships between foraminiferal assemblages and hydrodynamics in the Gulf of Kalloni, Greece. Journal of Foraminiferal Research 35: 327-343.

Debenay JP. 2012. A guide to 1000 foraminifera from South Western Pacific, New Caledonia. Institut de recherche pour le développement Marseille Publications Scientifiques du Muséum, Muséum national d’Histoire naturelle Paris.

de Blainville HMD. 1827. Manuel de malacologie et de conchyliologie. F. G. Levrault, Paris.

Defrance JLM. 1824. Dictionnnaire des Science Naturelles 32. F.G. Levrault, Strasbourg.

Delage Y, Herouard E. 1896. Traité de Zoologie Concrète, Vol. I, La Cellule et les Protozoaires. Schleicher Freres, Paris.

de Montfort D. 1808. Conchyliologie Systematique et Classification Methodique des Coquilles, Volume 1, F. Schoell, Paris.

Deželić V. 1896. Foraminifere Jadranskog mora. Glasnik Hrvatskog naravoslovnog drustva 9: 1-97.

d’Orbigny A. 1826a. Tableau Methodique de la Classe des Cephalopodes. Annales des Sciences Naturelles 7: 96-314.

d’Orbigny A. 1826b. Tableau Methodique de la Classe des Cephalopodes. Annales des Sciences Naturelles 7: 96-314.

d’Orbigny A. 1839a. Foraminiféres, p. 224. In: De la Sagra RM, ed. Histoire physique, politique et naturelle de L'ile de Cuba. Bertrand, A., Paris.

d’Orbigny A. 1839b. Foraminiféres, p. 119-146. In: Barker-Webb P, Berthelot S, eds. Histoire Naturelle des Iles Canaries, Volume 2, Part 2. Bethune, Paris.

d’Orbigny A. 1839c. Voyage dans I'Amerique Meridonale - Foraminiferes, part 5(5). Paris and Strasbourg.

d’Orbigny A. 1846. Foraminiferes Fossiles Du Bassin Tertiaire De Vienne (Autriche). Gide et Comp, Libraires Editeurs, Paris.

d’Orbigny A. 1852. Prodome de paléontologie stratigraphique universelle des animaux mollusques & rayonnés paisant suite au cours élémentaire de paléontologie et de géologie stratigraphiques, Vol 3. Victor Masson, Paris.

Dorreen JM. 1948. A foraminiferal fauna from the Kaiatan stage (upper Eocene) of New Zealand, Journal of Paleontology 22: 281-300.

Earland A. 1933. Foraminifera. Part 2. South Georgia Discovery Reports 7: 27-138.

Egger JG. 1893. Foraminiferen aus Meeresgrundproben, gelothet von 1874 bis 1876 von S.M. Sch. Gazelle. Abhandlungen der Bayrischen Akademie der Wissenschaften, Mathematisch-Physikalische Classe 18: 193-458.

Ehrenberg, CG. 1838. Über dem blossen Auge unsichtbare Kalkthierchen und Kieselthierschen als Hauptbestandtheile der Kreidegebirge. Bericht über die zur Bekanntmachung geeigneten Verhandlungen der Königlichen Preußischen Akademie der Wissenschaften zu Berlin 1838: 192-200.

Ehrenberg CG. 1839. Über die Bildung der Kreidefelsen und des Kreidemergels durch unsichtbare Organismen. Abhandlungen der Königlichen Akademie der Wissenschaften zu Berlin 1838: 60-148.

Ehrenberg CG. 1843. Verbreitung und Einfluß des mikroskopischen Lebens in Süd- und Nord-Amerika. Abhandlungen der Königlichen Akademie der Wissenschaften, Berlin 1841: 291-446.

Eimer GHT, Fickert C. 1899. Die Artbildung und Verwandtschaft bei den Foraminiferen. Entwurf einer natürlichen Eintheilung derselben. Zeitschrift für Wissenschaftliche Zoologie 65: 599-708.

Ellis BF, Messina A. 1940-2005. Catalogue of Foraminifera. The Micropaleontology Project, Inc., New York; http.micropress.org/em.

Fichtel Lv, Moll JPCv. 1798. Testacea microscopica, aliaque minuta ex generibus Argonauta et Nautilus, ad naturam picta et descripta (Microscopische and andere kleine Schalthiere aus den Geschlechtern Argonaute und Schiffer). Camesina, Wien.

Fiorini F. 2004. Benthic foraminiferal associations from upper Quaternary deposits of southeastern Po plain, Italy. Micropaleontology 50: 45-58.

Fleming J. 1828. A History of British Animals, Exhibiting the Descriptive Characters and Systematic Arrangement of the Genera and Species of Quadrupeds, Birds, Fishes, Moollusca and Radiata of the United Kingdom. Bell & Bradfute, Edinburgh.

Flint JM. 1899. Recent foraminifera, a descriptive catalogue of specimens dredged by the U.S. Fish Commision Streamer "Albatross". Report of the U.S. National Museum, Part I (1897): 249-349.

Fornasini C. 1901. Contributo a la conoscenza de le Bulimine Adriatiche. Memorie della Reale Accademia delle Scienze dell`Instituto di Bologna, Series 5 9: 371-381.

Forskal P. 1775. Descriptiones animalium. Hauniae. Carsten Niebuhr, Copenhagen.

Frezza V, Carboni MG. 2009. Distribution of recent foraminiferal assemblages near the Ombrone River mouth (Northern Tyrrhenian Sea, Italy). Revue de micropaleontologie 52: 43-66.

Fursenko AV. 1958. Osnovnye etapy razvitiya faun foraminifer v geologicheskom proshlom. Trudy Instituta Geologicheskikh Nauk, Akademiia Nauk Belomsskoi SSR, Minsk 1: 10-29.

Galloway JJ. 1933. A Manual of Foraminifera. Principal Press, Bloomington.

Glaessner MF. 1937. Die Entfaltung der Foraminiferenfamilie Buliminidae. Problemy Paleontologii, Paleontologicheskaya Laboratoriya Moskovskogo Gosudarstvennogo Universiteta 2-3: 411-422.

Golev BT. 1961. O rode Operculinoides Hanzawa. Voprosy Mikropaleo, S.S.S.R. 5: 112-120.

Gorbachik TN, Mantsurova VN. 1980. On the systematic position, rank and composition of the Spirillinidae (Foraminifera). Materialy VIII Mikropaleontologičeskogo Soveŝaniâ “Sistematika i Morfologiâ Mikroorganizmov 36-37.

Graham IJ, Militante PI. 1959. Recent foraminifera from the Puerto Galera area, northern Mindoro, Philippines, Stanford University Publications, Geological Sciences 6(2): 1-171.

Griffith JW, Henfrey A. 1875. The Micrographic Dictionary. Volume 1, 3rd Edition. Van Voorst, London.

Gronovius LT. 1781. Zoophylacium Gronovianum, vol. 3. Leyden, Thcodorus Haak et Society, 241-380.

Haeckel E. 1894. Systematische Phylogenie. Entwurf eines Natürlichen Systems der Organismen auf Grund ihrer Stammesgeschichte, Theil 1, Systematische Phylogenie der Protisten und Pflanzen. Georg Reimer, Berlin.

Hageman J. 1979. Benthic foraminiferal assemblages from Plio-Pleistocene open bay to lagoonal sediments of the western Peloponnesus (Greece). Utrecht Micropaleontological Bulletin 20: 1-171.

Hansen HJ, Lykke-Andersen AL. 1976. Wall structure and classification of fossil and recent elphidiid and nonionid foraminifera. Fossils and Strata 10: 1-37.

Hansen HJ, Reiss Z. 1972. Scanning Electron Microscopy of some Asterigerinid Foraminiferida. Journal of Foraminiferal Research 2(4): 191-199.

Hasegawa S, Sprovieri R, Poluzzi A. 1990. Quantitative analysis of benthic foraminiferal assemblages from Plio-Pleistocene sequences in the Tyrrhenian Sea, ODP LEG 107, p. 461-478. In: Kastens KA, Mascle J et al., eds. Proceedings of the Ocean Drilling Program, Scientific Results, Vol. 107. College Station, TX (Ocean Drilling Program), Texas.

Hatta A, Ujiie H. 1992. Benthic foraminifera from Coral Seas between Ishigaki and Iriomote Islands, Southern Ryukyu Island Arc, Northwestern Pasific. Bulletin of the College of Science, University of the Ryukyus 53: 49-119.

Hayward BW, Grenfell HR, Ried CM, Hayward KA. 1999. Recent New Zealand shallow-water benthic foraminifera: taxonomy, ecologic distribution, biogeography, and use in paleoenvironmental assessment. Institute of Geological and Nuclear Sciences Monographs 21: 1-264.

Hermelin JOR, Scott DB. 1985. Recent benthic foraminifera from the central North Atlantic. Micropaleontology 31: 199-220.

Heron-Allen, E. and Earland, A. 1915. The foraminifera of the Kerimba Archipelo (Portuguese East Africa), Part II. Transactions of the Zoological Society of London, 20, Part 17:543-794.

Heron-Allen E, Earland A. 1916. The foraminifera of the West of Scotland. Transactions of the Linnean Society of London, Series 2 11: 197-300.

Heron-Allen E, Earland A. 1930. The foraminifera of the Plymouth District. Journal of the Royal Microscopical Society, Series 3 50: 46-84.

Hoeglund H. 1947. Foraminifera in the Gullmar fjord and the Skagerak. Zoologiska Bidrag fran Uppsala 26: 3-328.

Hofker J. 1932. Notizen über die Foraminiferen des Golfes von Neapel III, Die Foraminiferenfauna des Ammontatura. Pubblicazioni della Stazione Zoologica di Napoli 12: 61-144.

Hofker J. 1951. The foraminifera of the Siboga Expedition, Part III. Siboga Expetitie, Monography IV, E.J. Bill, Leiden.

Hofker J. 1960. Foraminiferen aus dem Golf von Neapel. Paläontologische Zeitschrift 34: 233-262.

Hohenegger J, Piller W. 1975. Wandstrukturen und Grossgliederung der Foraminiferen. Sitzungsberichte der Österreichischen Akademie der Wissenschaften, Mathematisch-naturwissenschaftliche Klasse, Abteilung I 184(1-5): 67-96.

Holburn A, Henderson A, McLeod N. 2013. Atlas of Benthic Foraminifera. John Wiley and Sons. DOI: 10.1002/9781118452493

Hornibrook N de B, Vella P. 1954. Notes on the generic names of'some rotaliform foraminifera. Micropaleontologist 8(1): 24-28.

Hottinger L. 1977. Foraminiferes operculiniformes. Memoires du Museum National d'Histoire Naturelle, Series C, Sciences Terre 40: 1-159.

Hottinger L, Halicz E, Reiss Z. 1993. Recent foraminiferida from the Gulf of Aqaba, Red Sea. Academia Scientiarum et Artium Slovenica, Classis IV: Historia Naturalis, 33, Lubljana.

Jones TR, Parker WK. 1860. On the Rhizopodal fauna of the Mediterranean, compared with that of the Italien and some other Tertiary deposits. Quarterly Journal of the Royal Meteorological Society 16: 292-307.

Jones RW. 1994. The Challenger Foraminifera. Oxford University Press, Inc., New York.

Jorissen FJ. 1987. The distribution of benthic foraminifera in the Adriatic Sea. Marine Micropaleontology 12: 21-48.

Jorissen FJ. 1988. Benthic foraminifera from the Adriatic Sea; principles of phenotypic variations. Utrecht Micropaleontological Bulletin 37: 1-174.

Kaminski MA, Aksu A, Box M, Hiscott RN, Filipescu S, Al-Salameen M. 2002. Late Glacial to Holocene benthic foraminifera in the Marmara Sea: implications for Black Sea - Mediterranean Sea connections following the last deglaciation. Marine Geology 190: 162-202.

Kisel’man E.N. 1972. Verkhnemelovye i Paleotsenovye foraminifery novogo roda Spiroplectinella. Trudy Sibirskogo Nauchno-Issledovatel’skogo Instituta Geologii Geofizii i Mineralnogo Syrya (SNIIGGIMS) Ministerstva Geologii i Okhrany Nedr SSSR. Novosibirsk 146: 134-140.

Kruit C. 1955. Sediments of the Rhone Delta: Grain size and microfauna. Verhandelingen van het Koninklijk Nedarlandsch Geologisch Mijnbouwkundig Genootshap, Geologische Serie 15(3): 357-499.

Lacroix E. 1932. Textularidae du plateau continental mediterraneen entre Saint Raphael et Monaco. Bulletin de L´Institut Oceanographique de Monaco 591: 1-28.

Lalicker GC. 1935. Two new foraminifera of the genus Textularia. Smithsonian Institution Miscellaneous Collections, Washington 91(22): 1-2.

Lalicker GC, McCulloch I. 1940. Some Textulariidae of the Pacific Ocean. Allan Hancock Pacific Expeditions 6: 115-143.

Lamarck JB. 1804. Suite des memoires sur les fossiles des environs cle Paris. Annales Museum National d'Histoire Naturelle 5: 179-188.

Lankester ER. 1885. Protozoa. In: Encyclopaedia Britannica (9th ed, vol. 19). London, 830-866

Larsen AR. 1976. Studies of Recent Amphistegina, taxonomy and some ecological aspects. Israel Journal of Earth Sciences 26: 1-26.

Larsen AR, Drooger CW. 1977. Relative thickness of the test in the Amphistegina species of the Gulf of Elat. Utrecht Micropaleontological Bulletins 15: 225-239.

Lefoy LW. 1944. Miocene foraminifera from Sumatra and Java, Netherlands East Indies, Part. 1. Miocene foraminifera of central Sumatra, Netherlands East Indies. Colorado School of Mines Quarterly 39(3): 1-69.

Leiter C. 2008. Benthos-Foraminiferen in Extremhabitaten: Auswertung von METEOR-Expeditionen vor Namibia. Unpublished Ph.D. Thesis, Ludwig-Maximilians- Universität, München, Germany.

Le Calvez Y. 1949. Revision des foraminiferes Lutetiens du Bassin de Paris. II. Rotaliidae et families affines. Memoires du Service de la Carte Geologique Detaillee de la France, 1-54.

Le Calvez Y. 1958. Le foraminiferes de la Mer Celtique. Revue des Travaux de l'Institut des Peches Maritimes 22: 147-209.

Le Calvez Y. 1974. Revision des foraminiferes de la collection d'Orbigny. I-Foraminiferes des Iles Canaries. Cahiers de Micropaleontologie 1974(2): 1-108.

Le Calvez Y. 1977. Revision des foraminiferes de la collection d'Orbigny. 11. Foraminiferes de l'lle de Cuba-Tome 1. Cahiers de Micropaleontologie, 1977(1):1-128.

Le Calvez Y. 1977. Foraminiferes de l'fIe de Cuba- Tome 2. Cahiers de Micropaleontologie 1977(2): 1-131.

Le Calvez Y, Le Calvez J. 1958. Répartition des foraminiféres dans la Baie de Villefrance, I. Miliolidae. Annales de L'Institut Océanographique 35: 159-234.

Lehmann R. 1961. Strukturanalyse einiger Gattungen der Subfamilie Orbitolitinae, Eclogae Geologicae Helvetiae 54: 597-667.

LeRoy LW. 1941. Smaller Foraminifera from the Late Tertiary of the Netherlands East Indies, Part 2, Small foraminifera from the Late Tertiary of Siberoet Island, off the west coast of Sumatra, Nederlands East Indies. Colorado School of Mines Quarterly 36(1): 63-105.

Leroy LW. 1964. Smaller foraminifera from the late Tertiary of southern Okinawa, US Geological Survey Professional Paper 454: 1-58

Linné C. 1758. Systema naturae per regna tria naturae, secundum classes, ordines, genera, species, cum characteribus, differentiis, synonymis, locis, 1, 10th edition. L. Salvii, Holmiae (Stockholm).

Loeblich ARJr, Tappan H. 1955. Revision of some Recent foraminiferal genera. Smithsonian Miscellaneous Collections 10: 136-142.

Loeblich ARJr, Tappan H. 1957. Eleven new genera of foraminifera. Bulletin of the United States National Museum 215: 223-232.

Loeblich ARJr, Tappan H. 1961. Supragenetic classification of the Rhizopodea. Journal of Paleontology 35: 245-330.

Loeblich ARJr, Tappan H. 1964. Sarcodina chiefly “Thecamoebians” and Foraminiferida. In: Moore RC, ed. Treatise on Invertebrate Paleontology, Part C. Protista 2. Lawrence, Geological Society of America and University of Kansas Press.

Loeblich ARJr, Tappan H. 1981. Suprageneric revisions of some calcareous Foraminiferida. Journal of Foraminiferal Research 11: 159-164.

Loeblich ARJr, Tappan H. 1987. Foraminiferal genera and their classification. Von Nostrand Reinhold Co., New York.

Loeblich ARJr, Tappan H. 1994. Foraminifera of the Sahul Shelf and Timor Sea. Special Publication of the Cushman Foundation for Foraminiferal Research Special Publication 31: 1-661.

Luczkowska E. 1972. Miliolidae (Foraminiferida) from Miocene of Poland Part I. Revision of the classification. Acta Paleontologica Polonica 17: 341-377.

Luczkowska E. 1974. Miliolilidae (Foraminiferida) from the Miocene of Poland. Part II. Biostratigraphy, palaeoecology and systematics. Acta Palaeontologica Polonica 19: 3-176.

Mayer EM. 1968. Subclass Foraminifera. In: Atlas of invertebrates of the Caspian Sea. Kasp. NIRH. UNIRO. Moskva, 12-38.

Maync W 1952. Critical taxonomic study and nomenclatural revision of the Lituolidae based upon the prototype of the family Lituola nautiloidea Lamarck, 1804. Contributions from the Cushman Foundation for Foraminiferal Research 3: 35-56.

McCulloch L. 1977. Qualitative Observations on Recent Foraminiferal Tests with Emphasis on the Eastern Pacific: Parts I-III. University of Southern California, Los Angeles.

Mehrnusch M. 1993. Morphologische und strukturelle Merkmale einiger Bolivinen (Foraminiferida). Diskussion des taxonomischen Status von Afrobulimina, Brizalina, Bolivina and verwandten Taxa. Paläontologische Zeitschrift 67: 3-19.

Mendes I, Gonzalez R, Dias JMA, Lobo F, Martins V. 2004. Factors influencing recent benthic foraminifera distribution on the Guandiana shelf (Southwestern Iberia). Marine Micropaleontology 51: 171-192.

Mikhalevich VI. 1980. Novoe podsemeystvo Discammininae Mikhalevich, subfam. n. (Lituolidae, Foraminifera). In: Novoe v Sistematike Morskikh Bespozvochnykh Issledovaniya Fauny Morey. Zoologicheskiy Institut, Akademiya Nauk SSR, 5-7. (In Russian)

Milker Y. 2010. Western Mediterranean shelf foraminifera: Recent distribution, Holocene sea-level reconstructions, and paleoceanographic implications. Unpublished Ph.D. Thesis, University of Hamburg, Hamburg, Germany. Access via [http://ediss.sub.unihamburg](http://ediss.sub.unihamburg/). de/volltexte/2010/4709/

Milker Y, Schmiedl G, Betzler C, Römer M, Jaramillo-Vogel D, Siccha M. 2009. Distribution of Recent benthic foraminifera in neritic carbonate environments of the Western Mediterranean Sea. Marine Micropalaeontology 73: 207-225.

Milker Y, Schmiedl G. 2012. A taxonomic guide to modern benthic shelf foraminifera of the western Mediterranean Sea. Palaeontologia electronica 15(2): 1-134.

Millet FW. 1899. Report on the recens Foraminifera of the Malay Archipelago collected by Mr. A. Durand. F.R.M.S. part VI. Journal of the Royal Microscopical Society 20: 607-614.

Moebius KA. 1880. Foraminifera von Mauritius, p. 65- 112. In: Moebius KA et al., eds. Beiträge zur Meeresfauna der Insel Mauritius und der Seychellen. Gutman, Berlin.

Montagu G. 1803. Testacea Brittanica or Natural History of British Shells Marine, Land and Fresh Water, Including the Most Minute. J.S. Hollis, Romsey.

Montfort D. 1808. Conchyliologie Systematique et Classification Methodique des coquilles. F. Schoell, Paris, 1-409.

Morariu A, Hottinger L. 1988. Amphisteginids: specific identification, dimorphism, coiling direction and provincialism. Revue de Paléobiologie 2: 695-698.

Murray JW. 1970. Foraminifers of the Western Approaches to the English Channel. Micropaleontology 16: 471-85.

Murray JW. 1971. An Atlas of British Recent Foraminiferids. Heinemann Educational Books, London.

Murray JW. 2003. An illustrated guide to the benthic foraminifera of the Hebridean shelf, west of Scotland, with notes on their mode of life. Paleaeontologia Electronica 5(1): 1-31

Papp, A., Schmid, ME. 1985. Die fossilen Foraminiferen des Tertiaren Beckens von Wien. Revision der Monographie von Alcide d’Orbigny (1846). Geologischen Bundesanstalt, Wien.

Parker FL. 1952. Foraminifera species off Portsmouth, New Hampshire. Bulletin of the Museum of Comparative Zoology at Harvard College 106(9): 391-423.

Parker EL. 1954. Distribution of the foraminifera in the northeastern Gulf of Mexico. Bulletin of the Museum of Comparative Zoology at Harvard College 111(10): 453-588.

Parker FL. 1958. Eastern Mediterranean Foraminifera. Reports of the Swedish Deep-Sea Expedition, Sediment Cores from the Mediterranean Sea and the Red Sea 8: 217-285.

Parker JH. 2009. Taxonomy of Foraminifera from Ningaloo Reef, Western Australia. Association of Australasian Palaeontologists, Memoir 36.

Parker WK, Jones TR. 1859. On the nomendatureofthe foraminifera Part II. On the species enumerated by Walker and Montagu. Annals and Magazine of Natural History 34: 333-351.

Parker WK, Jones TR. 1865. On some foraminifera from the North Atlantic and Arctic Oceans, including Davis straits and Baffin's Bay. Philosophical Transactions of the Royal Society of London 155: 325-441.

Parr WJ. 1950. Foraminifera. Reports B.A.N.Z. Antarctic Research Expedition 1929-1931, Series B (Zoology, Botany) 5(6): 232-392.

Perelis L, Reiss Z. 1975. Cibicididae in recent sediments from the Gulf of Elat. Israel Journal of Earth- Sciences 24: 73-96.

Patterson RT. 1985. Abditodentrix, a new foraminiferal genus in family Bolivinitidae, Journal of Foraminiferal Research 15: 138-140.

Philippi RA. 1844. *Enumeratio molluscorum siciliae cum viventium tum in tellure tertiaria fossilium*. Anton, E., Halis Saxonum (Halle).

Piveteau J. 1952. Traité de Paléontologie, Volume 1. Masson et Cie, Paris.

Podobina VM. 1978. Systematics and Phylogeny Haplofragmiida. Tomsk Stat University Press. (In Russian)

Rasmussen TL. 1991. Benthonic and planktonic foraminifera in relation to the early Holocene stagnation in the Ionian Basin, central Mediterranean. BOREAS 20: 357-376.

Rasmussen TL. 2005. Systematic paleontology and ecology of benthic foraminifera from the Plio-Pleistoene Kallithea Bay section, Rhodes, Greece. Cushman Foundation Special Publication 39: 53-157.

Reiss Z, 1960. Structure of so-called Eponides and some other rotaliiform foraminifera. Bulletin of the Geological Survey of Israel 29: 1-28.

Reiss Z. 1963. Reclassification of perforate foraminifera. Bulletin of the Geological Survey of Israel 35: 1-111.

Reiss Z, Hottinger L. 1984. The Gulf of Aqaba. Ecological Micropalaeontology. Springer Science and Business Media.

Renz HH. 1948. Stratigraphy and fauna of the Agua Salada group, State of Falcon, Venezuela. Geological Society of America.

Resig JM. 2004. Age and preservation of Amphistegina (foraminifera) in Hawaiian beach sand: implication for sand turnover rate and resource renewal. Marine Micropaleontology 50(3-4): 225-236.

Resig JM. 1981, Biogeography of benthic foraminifera of the northern Nazca plate and adjacent continental margin. Memoirs of of the Geological Society of America 154: 619-665.

Reuss AE. 1850. Neue Foraminiferen aus den Schichten des österreichischen Tertiärbeckens. Denkschriften der mathematisch-naturwissenschaftlichen Classe der kaiserlichen Akademie der Wissenschaften 1849(1): 360-395.

Reuss AE. 1862. Entwurf einer systematischen Zusammenstellung der Foraminiferen. Sitzungsberichte der Kaiserlichen Akademie der Wissenschaften in Wien, Mathematisch-Naturwissenschaftliche Classe (1861) 44(1): 355-396.

Reuss AE. 1851. Ueber die fossilen Foraminiferen und Entomostraceen del' Septarienthone del' Umgegend von Berlin. Zeitschnt der Delltschen Geologischen Gesellschafl, 3: 49-91.

Reuss AE. 1851. Die Foraminiferen und Entomostraceen des Kreidemergels von Lemberg. Natuwiissenschaftliche Abhandlungen, 4: 17-52.

Reuss AE, Fritsch A. 1861. Verzeichniss von 100 Gypsmodellen von Foraminiferen, welche unter der Leitung des Prof. Dr. A. Reuss und Dr. Anton Fritsch gearbeitet wurden. Karl Seyfried, Prague.

Rhumbler L. 1895. Entwurf eines naturlichen Systems der Thalamophoren. Nachrichten von del Gesellschaft der Wissenschaften zu Gottingen. Mathematisch-Physikalische Klasse 1895(1): 51-98.

Rhumbler L. 1906. Foraminiferen von Laysan und den Chatham-Inseln. Zoologischer Jahresbericht 24: 21- 80.

Rhumbler L. 1911. Die Foraminiferen (Thalamophoren) der Plankton-Expedition, Erster Teil, Die AllgemeinenOrganizationsverhältnisse der Foraminiferen. Ergebnisse der Planktonexpedition der Humboldt-Stiftung Kiel und Leipzig, Band 3 L.c. (1909): 1-331.

Riveiros NV, Patterson RT. 2008. An illustrated guide to Fjord foraminifera from the Seymour-Belize inlet complex, Northern British Columbia, Canada. Palaeontologia Electronica 11(1): 2A, 45p. http://palaeo-electronica.org/2008_1/145/index.html.

Rogl E, Hansen HJ. 1984. Foraminifera described by Fichtel & Moll in 1798. A revision of Testacea Microscopica. Appendix Testacea Microscopica alique minuta ex Generibus Argonauta et Nautilus. Reprint of original plates. Neue Denkschnften des Naturhistorischen Museum in Wien 3: 1-143.

Rosset-Moulinier M. 1972. Etudes des Foraminifères des côtes nord et ouest de Bretagne. PhD Thesis, University of Paris.

Rzehak A. 1885, Bemerkungen über einige Foraminiferen der Oligozän Formation. Verhandlungen des Naturforschenden Vereins in Brünn 23: 123-129.

Rzehak A. 1888. Die Foraminiferen des kieseligen Kalkes von Nieder-Hollabrunn und des Melettamergels der Umgebung von Bruderndorf in Niederosterreich. Annalen NaturhislOrisches Hofmuseum. Wien 3: 257-270.

Said R. 1949. Foraminifera of the northern Red Sea, Special Publicalions Cushman Laboratory for Foramimjeral Research 26: 1-44.

Saidova KhM. 1981. O sovremennom sostoyanii sistemi nadvidovykh taksonov Kaynozoyskikh bentosnykh foraminifer. Institut Okeanologii P.P. Shirshova, Akademiya Nauk SSSR, Moscow. (In Russian)

Saidova KhM. 1975. Bentosnye Foraminifery Tikhogo Okeana, 3. Vol. Institut Okeanologii P.P. Shirshova, Akademiya Nauk SSSR, Moscow. (In Russian)

Schiebel R. 1992. Rezente benthische Foraminiferen in Sedimenten des Schelfes und oberen Kontinentalhanges im Golf von Guinea (Westafrika): Recent benthic foraminifera in sediments of the shelf and upper continental slope from the Gulf of Guinea (West Africa). Berichte-Reports, Universität Kiel 51: 1-126.

Schönfeld J., Golikova E., Korsun S and Spezzaferri S. 2013. The Helgoland Experiment - assessing the influence of methodologies on Recent benthic foraminiferal assemblage composition. Journal of Micropaleontology, 32, 161-182, doi: 10.1144/jmpaleo2012-022.

Schlumberger C. 1882. Foraminiferes. In: Milne-Edwards A, ed. Rapport sur les travaux de la Commission chargee d’étudier la faune sous-marine dans les grandes profondeurs de la Méediterranée et de l’Ocean Atlantique. *Archives des missions scientifiques et littéraires*, Paris, 1-59.

Schlumberger C. 1893. Monographie des Miliolidees du Golfe de Marseille. Memoires de la Societé Zoologique de France, 6: 57-80.

Schröder CJ. 1986. Deep-water arenaceous Foraminifera in the northwest Atlantic Ocean. Geological Survey of Canda, Bedford Institute of Oceanography, Atlantic Geosciences Centre 71: 1–191.

Schultze MS. 1854. Über den Organismus der Polythalamien (Foraminiferen) nebst Bemerkungen über die Rhizopoden im Allgemeinen. Leipzig.

Schulze FE. 1875. Zoologische Ergebnisse der Nordseefahrt vom 21. Juli bis 9. September 1872, I Rhizopoden. 2. Jahresberichte der Kommission zur Untersuchung der deutschen Meere in Kiel 1874: 99-114.

Schwager C. 1876. Saggio di una classificazione dei foraminiferi avuto riguardo alle lore famiglie naturali. Bolletino R. Comitato Geologico d’Italia 7: 475-485.

Schwager C. 1877. Quadro del proposto sistema di classificazione dei foraminiferi con guscio. Bolletino R. Comitato Geologico d’Italia 8: 18-27.

Seguenza G. 1862. Dei terreni Terziarii del distretto di Messina, Parte II, Descrizione dei foraminiferi monothalamici delle marne Mioceniche del distretto di Messina. Capra, T., Messina.

Seiglie GA. 1965. Some observations on Recent, foraminifers from Venezuela, Part 1, Contributions of the Cushman Foundation on ForamimIeral Research 16: 70-73.

Seiglie GA. 1965. Notas sobre las familias Pegidiidae y Siphoninidae (Foraminiferida), genero y especies nuevos. Caribbean Journal of Science 5: 9-13.

Sellier de Civrieux J.M. 1977. Las Discorbidae del Mar Caribe, frente a Venezuela. Cuadernos Oceanográficos, Universidad de Oriente, Cumana 6: 1-44.

Sgarrella F, Moncharmont Zei M. 1993. Benthic foraminifera in the Gulf of Naples (Italy): systematics and autoecology. Bollettino della Societá Paleontologica Italiana 32: 145-264.

Shchedrina ZG, Mayer EM. 1975. About different forms of Ammonia beccarii (Linne). Complex investigations of oceans nature, part 5-Moskovskogo Univ. Izdat, 249-261, pl. 1-4. (In Russian)

Sidebottom H. 1905. Report on the Recent foraminifera from the coast of the Island of Delos

(Grecian Archipelago), Part II. Memoirs and Proceedings of the Manchester Literary and Philosophical Society 49(5): 1-22.

Silvestri A. 1923. Lo stipite della Elissoforme e Ie sue affinita, Memorie della Pontificia Accademia della Scienze, Nllovi Lincei 26: 231-270.

Smith RK, Isham LB. 1974. Reinstatement of Michostomina Berthelin, 1881, and emendation of Spirillina Ehrenberg. 1843. Spirillininae, Spirillinidae, and Spirillinacea, all Reuss, 1862. Journal of Foraminiferal Research 4: 61-68.

Souaya FJ. 1965. Miocene foraminifera of the Gulf of Suez region, U.A.R. Part 1: Systematics (Astrohizoidea: Buliminoidea). Micropaleontology 11: 301-334.

Spezzaferri S, Rüggeberg A, Stalder C., Margreth S. 2015. Systematic description and illustration of Benthic and planktonic foraminifera from cold water coral ecosystems. In: Spezzaferri S, Stalder C, Rüggeberg A, eds. Atlas of benthic foraminifera from cold water coral reefs. *Cushman Foundation Special Publication Series*, 249-140.

Sprovieri R, Hasegawa S. 1990. Plio-Pleistocene benthic foraminifer stratigraphic distribution in the Deep-Sea record of the Tyrrhenien Sea (OPD Leg 107). In: Kastens KA, Mascle J, eds. Proceedings of the Ocean Drilling Program, Scientific Results, Vol. 107. College Station, TX (Ocean Drilling Program), Texas, 429-459.

Sulc J. 1936. Etudes sur quelques genres et especes de Peneroplides, Annales de Protistologie 5: 157-170.

[Tappan](http://taxonomicon.taxonomy.nl/Person.aspx?id=1814) H, [Loeblich ARJr.](http://taxonomicon.taxonomy.nl/Person.aspx?id=433) 1982. Granuloreticulosa. [In Parker SP, ed. Synopsis and Classification of Living Organisms, vol. 1. McGraw-Hill, New York](http://taxonomicon.taxonomy.nl/Reference.aspx?id=1026), 527-552.

Terquem O, 1876. Essai sur Ie classement des animaux qui vivent sur la plage et dans les environs de Dunquerque, deuxieme fascicule. Paris, p. 55-100.

Terquem O. 1878. Les Foraminiféres et les Entomostracés-Ostracodes du Pliocène Supérieur de L'ile de Rhodes, Premiére Section-Foraminiféres. Memoires de la Societe Geologique de France Series 3(3):1-135.

Todd R. 1958. Foraminifera from the Western Mediterranean deep-sea cores. Reports of the Swedish Deep-Sea Expedition, Sediment Cores from the Mediterranean Sea and the Red Sea 8: 167-215.

Todd R. 1965. The foraminifera of the tropical Pacific collections of the "Albatross", 1899-1900, Part 4, Rotaliform families and planktonic families. United States National Museum Bulletin 161: 1-139.

van Marle L. 1991. Eastern Indonesian Late Cenozoic smaller benthic foraminifera. North-Holland, New York.

Vella P. 1957. Studies in New Zealand foraminifera. Paleontological Bulletin, Wellington, 28: 1-64.

Vénec-Peyré MT. 1984. Étude de la distribution des foraminifèresvivant dans la baie de Banyuls-sur-Mer. In: Bizon JJ, Burollet PF, eds. Ecologie des microorganismes en Méditerranée occidentale (ECOMED). Association française des Techniciens du Pétrole, Paris, 60-80.

Voloshinova NA. 1960. Uspekhi mikropaleontologii vdele izucheniya vnutrennego stroeniya foraminifer. In: Trudy Pervogo Seminara po Mikrofaune. Vsesoyuznyy Neftyanoy Nauchno-issledovatel’skii Geologorazvedochnyy Institut (VNIGRI), Leningrad, 48-87. (In Russian)

Voloshinova NA, Dain LG. 1952. Nonionidy. Kassidulinidy i Khilostomellidy. VNIGRI Proceedings, 63.

Walker G, Jacob E. 1798. In: Kanmacher F, ed. Adam`s Essay on the Microscope. Dillon and Keating, London.

Wenger WF. 1987. Die Foraminiferen des Miozänsder bayerischen Molasse und ihre stratigraphische sowiepaläogeographische Auswertung. Zitteliana 16: 173-340.

Wiesner H. 1920. Zur Systematik der Miliolideen. Zoologischer Anzeiger 51: 13-20.

Wiesner H. 1923. Die Miliolideen der östlichen Adria. The author, Prag-Bubanec.

Wiesner H. 1931. Die Foraminiferen der deutschen Südpolar- Expedition 1901-1903. In: von Drygalski E, ed, Deutsche Südpolarexpedition 1901-1903 (Volume 20). Zoologie 12, Walter de Gruyter and Co., Berlin und Leipzig, 49-165.

Williamson WC. 1852. On the minute structure of the calcareous shells of some recent species of foraminifer. Transactions of the Microscopical Society of London 3(1): 105-128.

Williamson WC. 1858. On the Recent foraminifera of Great Britain. Ray Society, London.

Wollenburg J. 1992. Zur Taxonomie von rezenten benthischen Foraminiferen aus dem Nansen Becken, Arktischer Ozean. Berichte zur Polarforschung 112: 1-137.

Yanko V. 1989. Chetvertichnie bentosnye foraminiferi Ponto-Kaspiia (Chernoe, Azovskoe,

Kaspiiskoe i Aral’skoe moria): taxonomiia, biostratigrafiia, istoriia razvitia, ekologiia

Quaternary Benthic Foraminifera of the Pontic-Caspian Region (Black, Azov, Caspian, and

Aral seas) seas: Taxonomy Biostratigraphy, History, Ecology. PhD Thesis, Moscow State

University, Moscow. (In Russian)

Zaouali J. 1993. Les peuplements benthiques de la petite Syrte, golfe de Gabès-Tunisie. Résultats de la campagne de prospection du mois de juillet 1990. Marine Life 3(1-2): 47-60.

Zheng SY. 1988. The Agglutinated and Porcelanaceous Foraminifera of the East China Sea. Science Press, Beijing
